# A Cross-Sectional Study of Nutrition Knowledge, Diet Quality, Lifestyle, and Health Profiles Among Older Adults Attending Universities of the Third Age in Poland

**DOI:** 10.3390/nu18122025

**Published:** 2026-06-22

**Authors:** Anna Miller, Agata Kotowska, Sabina Lachowicz-Wiśniewska

**Affiliations:** 1 Doctoral School, The Faculty of Medicine and Health Science, University of Kalisz, W. Bogusławskiego 2, 62-800 Kalisz, Poland; szd.5.2023@uniwersytetkaliski.edu.pl; 2 Institute of Sociology, University of Rzeszów, Al. T. Rejtana 16C, 35-959 Rzeszów, Poland; 3 Department of Nutrition and Food, The Faculty of Medicine and Health Science, University of Kalisz, W. Bogusławskiego 2, 62-800 Kalisz, Poland; 4 Department of Biotechnology and Food Analysis, Wroclaw University of Economics and Business, Komandorska 118/120, 53-345 Wroclaw, Poland

**Keywords:** older adults, healthy ageing, nutrition knowledge, diet quality, Diet Quality Index, Universities of the Third Age, lifestyle, multimorbidity, dietary patterns, nutrition education

## Abstract

**Background:** Population ageing increases the burden of chronic diseases, multimorbidity, and functional limitations, making nutrition and lifestyle important modifiable determinants of healthy ageing. Universities of the Third Age (U3A) provide an educational and social environment for older adults, but multidimensional relationships between nutrition knowledge, diet quality, lifestyle, and health status in this population remain insufficiently characterized. This study aimed to assess these associations among older adults attending U3A in Poland. **Methodology:** A cross-sectional online survey was conducted between January and April 2026 among community-dwelling older adults participating in U3A programs. Of 700 distributed invitations and 520 returned questionnaires, 450 complete and eligible responses were included. The questionnaire was based on the KomPAN^®^ framework and expanded with items on health, lifestyle, psychosocial resources, barriers to healthy eating, and sources of health information. Diet quality was assessed using the pro-Healthy Diet Index, non-Healthy Diet Index, and overall Diet Quality Index (DQI). Nutrition knowledge was measured using a 24-item scale. Analyses included distributional diagnostics, non-parametric group comparisons, FDR-corrected Spearman correlations, psychometric assessment, principal component analysis, multivariable regression with model diagnostics, and profile segmentation. **Results:** The mean age was 73.63 ± 5.73 years, and most participants were women. The median DQI was 15.59 [3.93–24.86], with a predominance of neutral diet quality. Nutrition knowledge was moderate, with a median score of 12.00 [9.00–15.00], and the scale showed very good internal consistency. PCA identified three dietary patterns: convenience/ultra-processed, prudent/health-promoting, and traditional meat-and-fat. Higher DQI was associated with better nutrition knowledge, greater physical activity, a more favorable sleep profile, regular meal timing, and lower disease burden. Participants with multimorbidity had significantly lower DQI. Segmentation distinguished a health-engaged/higher-resource profile and a lower-resource/nutritionally vulnerable profile. **Conclusions:** U3A participants in Poland are educationally and socially active but nutritionally heterogeneous. The predominance of neutral diet quality, moderate nutrition knowledge, and identifiable knowledge gaps indicates the need for targeted, practical, and behavior-oriented nutrition education supporting healthy ageing.

## 1. Introduction

Population ageing represents one of the major challenges of contemporary societies, generating multidimensional consequences for healthcare systems, social policy, and public-health planning [1]. This process reflects not only changes in life expectancy, but also long-term demographic shifts, including declining fertility, cohort ageing, and the increasing proportion of older adults in the total population. Globally, the number of people aged 60 years and older is expected to double by 2050, reaching 2.1 billion, while the population aged 80 years and older is projected to nearly triple between 2020 and 2050, reaching 426 million [2]. In Poland, the ageing process is also progressing rapidly. According to the 2024 report of the Ministry for Senior Policy, the number of people aged 60 years and older has reached nearly 10 million, increasing by 85.3 thousand compared with 2023, and older adults now account for 26.6% of the Polish population. Particularly dynamic growth has been observed in the 75–79-year age group [3]. Population forecasts prepared by Statistics Poland indicate that this trend will continue, with the proportion of people aged 60 years and older expected to approach approximately 40% of the total population by 2060 [4].

At the same time, the increasing proportion of older adults should be interpreted alongside recent unfavorable trends in life expectancy and healthy life years in Poland. After several decades of improvement, gains in life expectancy slowed markedly in the mid-2010s, and the COVID-19 pandemic further intensified this negative trend through excess mortality. Although life expectancy increased nominally after the pandemic period, healthy life years have not improved at a comparable pace, indicating that many older adults spend a substantial proportion of later life with chronic diseases, multimorbidity, or functional limitations. In 2023, life expectancy in Poland remained lower than the European Union average, particularly among men, and healthy life years at age 65 were also less favorable than the EU average. Thus, population ageing in Poland should be understood not simply as longer survival, but as an increasing demographic and public-health burden associated with chronic disease, disability risk, and the need to support healthy ageing [5]. In 2024, the leading causes of death among older adults in Poland were cardiovascular diseases, accounting for 39.9% of deaths, and cancers, accounting for 27% [6].

Health perception among older adults is also an important contextual factor. Older people do not always interpret health as a consequence of lifestyle, but rather as a state of relative functional ability or the absence of evident symptoms [7]. According to 2024 data, 47.2% of Polish seniors rated their health as “neither good nor poor”, 30.9% as “good”, and only 2.2% as “very good”; men assessed their health more favorably than women [6]. At the same time, population ageing increases the prevalence of chronic diseases and multimorbidity, contributing to the growing burden of non-communicable diseases and to the widespread use of polypharmacy among older adults [8,9]. These processes are substantially influenced by modifiable lifestyle factors, which play a key role in both the development and progression of chronic conditions [10,11].

Age-related physiological and psychosocial changes should be considered when planning nutrition strategies for older adults, as they influence nutritional needs, dietary choices, and the ability to maintain health-promoting behaviours [12,13].

Proper nutrition is one of the foundations of healthy ageing. In older adults, physiological changes, including altered body composition, impaired gastrointestinal function, reduced sensory sensitivity, and changes in the regulation of energy metabolism, interact with behavioral and socioeconomic determinants, directly influencing dietary patterns and nutritional status [14]. Population data indicate that physical inactivity combined with lower diet quality may contribute to the development and progression of chronic diseases, including cardiovascular diseases, metabolic disorders, obesity, and type 2 diabetes, as well as to functional decline, frailty, and reduced quality of life. Conversely, adherence to health-promoting dietary patterns is associated with a more favorable cardiometabolic profile, preservation of cognitive function, and lower all-cause mortality [15,16,17].

A key priority in nutritional gerontology is therefore not only to describe dietary behaviors among older adults, but also to identify the personal, behavioral, and socioeconomic factors that shape these behaviors. Older adults constitute a population particularly vulnerable to nutritional irregularities, largely due to functional limitations, comorbidities, metabolic changes, and psychosocial factors such as loneliness, long-established habits, and dietary preferences [8]. Nutritional education is thus an essential component of strategies supporting healthy ageing, as it may help prevent dietary irregularities and contribute to improved health status and quality of life in later life.

Universities of the Third Age provide a particularly relevant setting for educational and health-promoting activities among older adults by integrating educational, social, and activation-oriented functions. Participation in such initiatives is associated with higher levels of cognitive and social activity, which may indirectly support the development of health-promoting behaviors, including healthier dietary patterns. However, the relationships between nutrition knowledge, diet quality, lifestyle, and health status among older adults should be considered within a broader psychosocial context that includes sources of health information, barriers to healthy eating, and patterns of activity and recovery. Such an approach reflects the multifactorial nature of health behavior determinants in this population.

Therefore, the aim of the present study was to assess multidimensional associations between nutrition knowledge, diet quality, lifestyle, and selected health indicators in a population of older adults attending Universities of the Third Age in Poland.

## 2. Materials and Methods

### 2.1. Study Design and Participants

This study was designed as a cross-sectional survey conducted among older adults attending Universities of the Third Age in Poland. Data were collected between January and April 2026. The study population comprised community-dwelling seniors participating in educational and social programs offered by Universities of the Third Age, which constituted the sole target population of the present analysis.

Participants were recruited through collaborating Universities of the Third Age and their local coordinators, who distributed an invitation to participate together with a link to the online questionnaire. Eligibility criteria included age of 60 years or older, current participation in a University of the Third Age program, ability to complete the questionnaire independently in an online format, and provision of informed consent. Only fully completed questionnaires were included in the final analysis. Responses were excluded if informed consent was not provided, the questionnaire was incomplete, or the respondent did not belong to the target population.

The final analytical sample consisted of 450 respondents. Place of residence was classified according to national statistical categories into urban, small-town, and rural settings. A total of 700 survey invitations were distributed. Recruitment was conducted through collaborating Universities of the Third Age. First, invitations were sent by e-mail to the administrative offices of participating institutions, with two reminder e-mails issued during the recruitment period. The questionnaire link was then disseminated through the internal communication systems of the respective Universities of the Third Age. In total, 520 questionnaires were returned. Following data screening and the exclusion of incomplete or ineligible responses, 450 questionnaires were retained for the final analysis.

The study protocol was approved by the Bioethics Committee of the University of Kalisz (Approval no. KB-551/2022) and was conducted in accordance with the ethical principles of the Declaration of Helsinki [18]. Participation was voluntary and anonymous. Before entering the questionnaire, all respondents were informed about the purpose of the study, the anonymous handling of data, and their right to withdraw at any stage, and they provided informed consent electronically.

### 2.2. Research Tool and Data Collection

Data were collected using an online questionnaire administered via SurveyMonkey (SurveyMonkey Inc., San Mateo, CA, USA). The survey instrument was based on the KomPAN^®^ (Olsztyn, Poland) [19,20] questionnaire developed by the Committee of Human Nutrition Science of the Polish Academy of Sciences and its established methodological framework for assessing dietary habits, food-consumption frequency, and nutrition-related behaviors [19,20]. For the purposes of the present study, the questionnaire was adapted to an online format and expanded with additional items relevant to the health, lifestyle, and psychosocial functioning of older adults attending Universities of the Third Age.

The questionnaire included sections covering sociodemographic characteristics, including age, sex, education, and place of residence, as well as self-reported anthropometric data, including body height, body weight, and waist circumference. Body mass index (BMI) was calculated as body weight in kilograms divided by height in meters squared (kg/m^2^). BMI categories were then assigned accordingly. Waist-circumference data were additionally used to classify abdominal obesity status.

Health-related variables included self-rated health, self-rated physical activity, sleep duration, meal regularity, supplement use, and self-reported presence of chronic diseases. Disease count was calculated as the number of declared chronic conditions, and multimorbidity was defined as the coexistence of at least two diseases.

Dietary assessment was based on the habitual frequency of consumption of selected food groups. On this basis, three diet-quality indicators were derived in accordance with the KomPAN methodology: the pro-Healthy Diet Index (pHDI), the non-Healthy Diet Index (nHDI), and the overall Diet Quality Index (DQI) [19,20]. The same food-frequency variables were subsequently used for principal component analysis to identify broader dietary patterns.

The questionnaire also included additional items on daily lifestyle and self-care behaviors, rest and recovery patterns, barriers to healthy eating, perceived sources of health information, and selected psychosocial resources. To characterize nutritional awareness, a 24-item nutrition knowledge scale was applied. Each correct answer was scored as 1 point, whereas incorrect answers and “do not know” responses were scored as 0 points. The total nutrition knowledge score, therefore, ranged from 0 to 24, with higher scores indicating better nutrition knowledge.

In the extended analytical framework, composite or scaled variables were also constructed for physical activity, cognitive activity, psychosocial resources, sleep, active ageing, and meal regularity. These variables were used in correlation analyses, multivariable association models, interaction analyses, and senior-profile segmentation.

### 2.3. Construction of Composite Lifestyle and Resource Scores

Several composite variables were constructed to summarize lifestyle, activity, and psychosocial-resource domains used in the multivariable and segmentation analyses. The physical activity score reflected declared level and frequency of activity-related behaviours, with higher values indicating a more active lifestyle. The cognitive activity score summarized participation in mentally stimulating activities, including educational, reading, cultural, or other cognitively engaging behaviours. The psychosocial resources score was based on indicators of social contact, perceived support, self-care behaviours, and engagement in well-being-oriented activities, with higher scores reflecting greater psychosocial resources. The sleep score was derived from reported sleep duration and regularity of rest, with higher values indicating a more favorable sleep profile. The meal regularity score reflected the regularity of daily meal timing and the number of meals consumed. Finally, the active ageing score was constructed as an integrated standardized indicator combining physical, cognitive, psychosocial, and selected lifestyle-resource domains. In all composite indicators, higher scores denoted a more favorable profile. Because these scores were used primarily for analytical stratification and profile characterization, their construction and theoretical ranges are described in detail in Table S1.

### 2.4. Statistical Analysis

Descriptive statistics were used to characterize the study population and key study variables. Continuous variables were assessed for distributional properties using graphical inspection and formal normality diagnostics. Because several variables showed non-normal or skewed distributions, they are presented primarily as medians and interquartile ranges, with means and standard deviations reported additionally where relevant for comparability. Categorical variables are presented as counts and percentages.

The psychometric properties of the nutrition knowledge scale were assessed using internal-consistency and item-level diagnostics. Principal component analysis (PCA) with varimax rotation was used to identify major food-consumption patterns. Component retention was based on eigenvalues, scree-plot inspection, explained variance, and interpretability. Detailed PCA diagnostics and the full rotated loading matrix are provided in the Supplementary Materials.

Associations between diet quality and behavioral, psychosocial, and health-related variables were examined using Spearman’s rank correlations, with Benjamini–Hochberg false discovery rate correction for multiple testing. Between-group differences in Diet Quality Index (DQI) were assessed using non-parametric tests, including the Mann–Whitney U test and Kruskal–Wallis test, with appropriate effect-size measures and post hoc comparisons where applicable.

Multivariable associations with diet quality were examined using adjusted regression models. Continuous DQI was analyzed using ordinary least squares regression with robust standard errors when model diagnostics indicated heteroscedasticity. A complementary logistic regression model was used for higher diet quality, defined as the upper tertile of DQI. Additional exploratory models were constructed for abdominal obesity, multimorbidity, and supplement use. Model diagnostics and detailed supplementary analyses are provided in the Supplementary Materials.

Finally, segmentation analysis was used to identify profiles of older adults according to nutritional, behavioral, psychosocial, and health-related characteristics. The resulting profiles were compared descriptively and visualized using a heatmap. Statistical significance was assessed using two-sided tests, with *p* < 0.05 considered significant unless FDR-adjusted q values were applied.

## 3. Results

### 3.1. Characteristics of the Study Population

The study population comprised 450 older adults with a mean age of 73.63 ± 5.73 years. The sample was predominantly female, mostly urban, and characterized by a high proportion of participants with higher education (Table 1). Normal body weight and overweight were the most common BMI categories, although abdominal obesity was present in a considerable proportion of respondents. Self-rated health was most often described as good or average, and self-rated physical activity was usually moderate.

The declared disease burden was generally low, and multimorbidity was observed in a minority of respondents. However, the disease co-occurrence network indicated clustering of cardiometabolic and related conditions, with hypertension, diabetes, hypercholesterolemia, and cardiovascular disease forming the central cluster (Figure S1). Chronic obstructive pulmonary disease (COPD), obesity, osteoporosis, gastrointestinal disease, and metabolic dysfunction-associated steatotic liver disease (MASLD) were positioned more peripherally within the network.

### 3.2. Health Status, Lifestyle, and Dietary Behaviors

Daily lifestyle and dietary-behavior characteristics are summarized in Table S2, with extended distributions provided in Tables S3–S5. Most participants reported sleeping 7–8 h per night and consuming three or four meals per day, while regular meal timing was declared by the majority of respondents. Self-care and recovery behaviors were mainly activity- and socially oriented, with physical and mental activity, social contact, and regular health check-ups reported most frequently.

Between-meal choices were dominated by fruit and vegetables, whereas salty snacks and sweetened dairy desserts or beverages were reported much less often. Cooking practices were generally consistent with health-promoting food preparation, as boiling/steaming, raw or non-heat-treated foods, and stewing were used most commonly, while grilling and deep-fat frying were rare. Natural honey was the most frequently declared sweetening option, followed by white and brown/cane sugar. Supplement use was reported by more than half of the respondents, most often vitamin D and magnesium preparations. A small proportion also indicated limited opportunities for rest due to family responsibilities or continued professional activity (Table S3).

Supplement use was reported by more than half of the study population (Tables S2 and S5). The most commonly declared categories were vitamin D and magnesium, followed by omega-3/fish oil preparations and B vitamins, whereas the use of other supplements was less frequent.

### 3.3. Diet Quality

Overall diet quality in the study population was summarized primarily using medians and interquartile ranges because diet-quality indicators showed non-normal distributions. The median pHDI was 28.40 [19.90–36.40], the median nHDI was 12.21 [6.23–19.34], and the median DQI was 15.59 [3.93–24.86] (Table 2). Mean ± SD values are additionally reported for comparability with previous studies. When categorized into three levels, most participants were classified in the neutral DQI category, whereas approximately one-quarter were classified as having a healthy diet profile, and only a marginal proportion fell into the unhealthy category.

**Figure 1:**
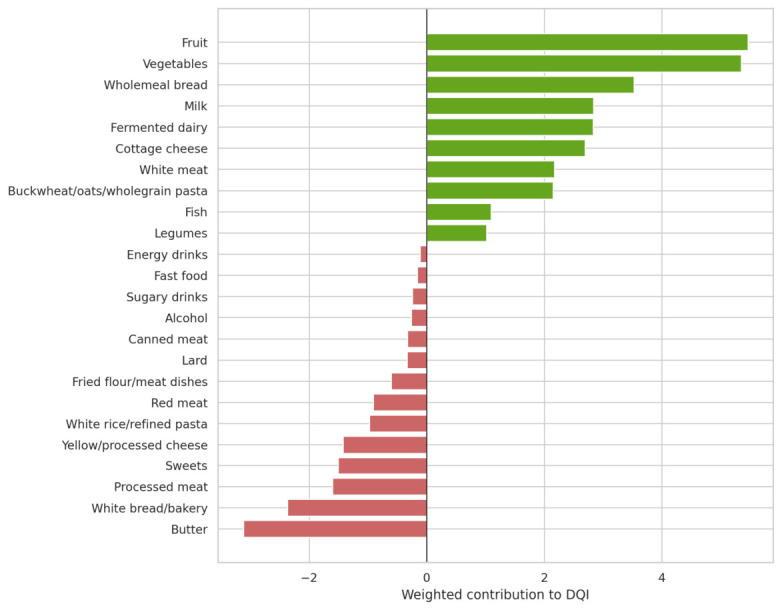
Food-group contribution to overall diet quality. Positive values indicate food groups contributing favorably to the Diet Quality Index (DQI), whereas negative values indicate food groups contributing unfavorably to the overall DQI score. Green bars represent favorable contributions to DQI, while red bars represent unfavorable contributions. The figure illustrates the relative direction and magnitude of the contribution of individual food groups to diet quality in the studied U3A population.

The food-group decomposition of overall diet quality showed that the strongest positive contributions to DQI were provided by fruit and vegetables, followed by wholemeal bread and selected dairy products, including milk, fermented dairy, and cottage cheese (Figure 1). Additional favorable contributions were observed for white meat, buckwheat/oats/wholegrain pasta, fish, and legumes. In contrast, the strongest negative contributions were associated with butter, white bread and bakery products, processed meat, sweets, and yellow/processed cheese. Smaller negative contributions were also observed for white rice/refined pasta, red meat, fried flour- or meat-based dishes, lard, canned meat, alcohol, sugary drinks, fast food, and energy drinks (Figure 1).

### 3.4. Dietary Patterns Identified by Principal Component Analysis (PCA)

Principal component analysis identified three major dietary patterns that jointly explained 38.62% of the total variance in food-consumption frequency (Table 3). The retained components had eigenvalues of 4.27, 3.28, and 2.13, respectively. Component retention was supported by eigenvalues greater than 1.0, inspection of the scree plot, explained variance, and interpretability of the loading structure (Figure S2). Varimax rotation was applied to improve interpretability. Food groups were assigned to dietary patterns based on absolute rotated loadings ≥ 0.30, with the final interpretation based on the highest absolute loading for each food group. The full rotated loading matrix is provided in Table S9.

The first dietary pattern explained 17.04% of the variance and was characterized by the highest positive loadings for energy drinks, sugar-sweetened beverages, canned meat, lard, and fast food. Based on the overall loading structure, this component was classified as a convenience/ultra-processed pattern. The second pattern explained 13.09% of the variance and was defined primarily by whole grains and groats, fish, white meat, fermented dairy drinks, and legumes, corresponding to a prudent/health-promoting pattern. The third pattern explained 8.49% of the variance and was driven mainly by processed meat, white bread, red meat, butter, and yellow cheese, and was therefore classified as a traditional meat-and-fat pattern (Table 3).

**Figure 2:**
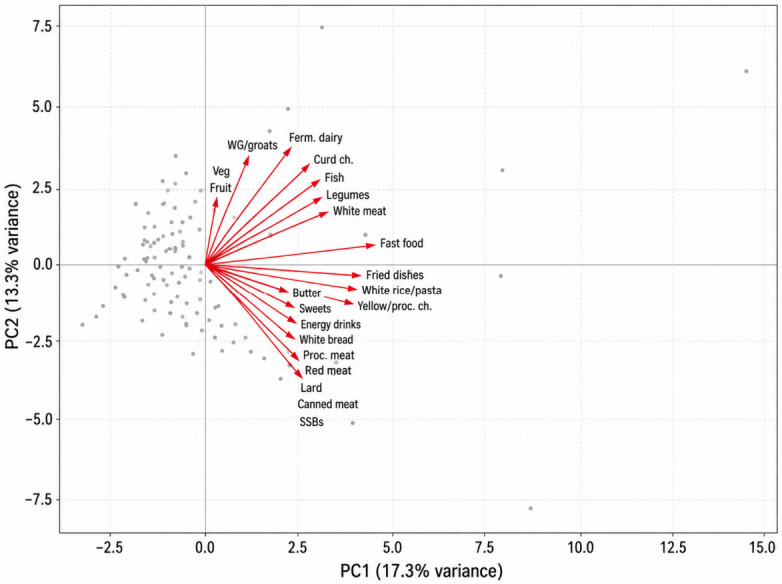
PCA biplot of food consumption patterns. The biplot shows the distribution of food groups in the space defined by the first two principal components. Food groups positioned in a similar direction are interpreted as contributing to a similar dietary pattern. Red arrows represent food-group loading vectors, whereas gray dots represent individual respondents projected onto the PC1–PC2 space. Abbreviated labels are used to improve readability; full food-group names and rotated component loadings are provided in Table S9.

The PCA biplot showed a clear separation of food groups across the first two components (Figure 2). Foods characteristic of the prudent/health-promoting pattern, including fruit, vegetables, wholegrain products, legumes, fish, white meat, and fermented dairy products, were positioned mainly in the upper part of the plot, indicating a shared direction of contribution to the second component. In contrast, foods such as sugar-sweetened beverages, canned meats, red meat dishes, processed meats, butter, sweets, and refined cereal products were distributed predominantly along the positive axis of the first component and in the lower part of the PC1–PC2 space. Fast food also showed a marked contribution in the positive direction of the first component.

The loadings heatmap further confirmed the structure of the retained components and showed consistent clustering of food groups within the identified patterns (Figures S3 and S4). Overall, the PCA distinguished three broad dietary profiles in the study population: one centered on convenience and ultra-processed products, one reflecting a more health-promoting food selection, and one characterized by the traditional meat-and-fat pattern.

### 3.5. Nutrition Knowledge and Psychometric Properties

The nutrition knowledge scale yielded a median score of 12.00 [9.00–15.00] points out of 24, with a mean ± SD value of 11.34 ± 4.93 reported for comparability, indicating a moderate overall level of performance in the study population (Table 4). The scale consisted of 24 items combined into a single summed score.

The psychometric evaluation demonstrated very good measurement properties of the instrument. Internal consistency was high, with a Cronbach’s alpha of 0.856, while the Kaiser–Meyer–Olkin measure reached 0.855, indicating very good sampling adequacy. Bartlett’s test of sphericity was statistically significant (χ^2^ = 2749.10, *df* = 276; *p* < 0.001), supporting the suitability of the item set for structure-based analysis (Table 4). Item-level diagnostics were also stable, with item-total correlations ranging from 0.159 to 0.520 and alpha-if-deleted values remaining within a narrow interval, indicating that removal of any single item did not materially improve overall scale reliability.

**Figure 3:**
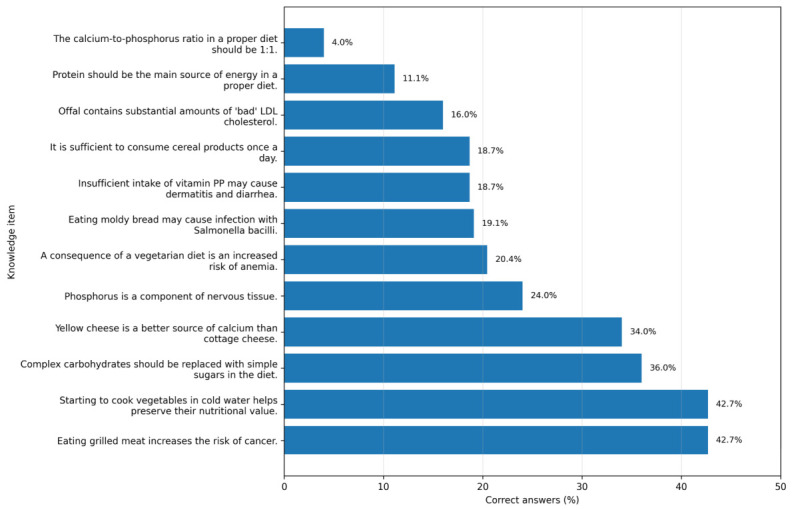
Nutrition knowledge gaps and item difficulty. The figure presents the proportion of correct and “do not know” responses for individual items of the 24-item nutrition knowledge scale. Items with lower proportions of correct answers and higher proportions of “do not know” responses indicate areas of greater knowledge deficit.

Considerable variation was observed across individual knowledge items (Figure 3; Table S7). The highest proportion of correct responses was recorded for the statement that sun exposure promotes endogenous vitamin D synthesis, followed by items concerning wholemeal bread as a richer source of fiber than refined bread, the beneficial role of bio-yogurts, reduction in fatty dishes in cardiovascular prevention, frequent consumption of fruit and vegetables, and the adverse effect of high salt intake on hypertension-related risk. In contrast, the lowest proportion of correct answers was observed for the item regarding the recommended calcium-to-phosphorus ratio in a healthy diet, which was also the item with the highest proportion of “do not know” responses. Low rates of correct responses were also noted for questions concerning vitamin PP deficiency, phosphorus as a component of nervous tissue, vegetarian diets and anemia risk, offal and LDL cholesterol, recommended cereal intake, and the role of protein as the main energy source in a healthy diet.

The pattern of item difficulty shown in Figure 3 confirmed that knowledge was unevenly distributed across thematic areas. Items related to vitamin D, fiber, fermented dairy products, salt, and dietary fat were answered correctly relatively often, whereas items addressing mineral balance, selected micronutrient deficiency symptoms, and several detailed nutrition facts showed substantially lower response accuracy.

### 3.6. Barriers, Sources of Knowledge, and Psychosocial Determinants of Health

The most frequently reported barrier to healthy eating was long-standing eating habits, followed by insufficient nutrition knowledge, low motivation, and financial constraints (Figure S5). Barriers related to time requirements or limited access to reliable information were reported less often.

**Figure 4:**
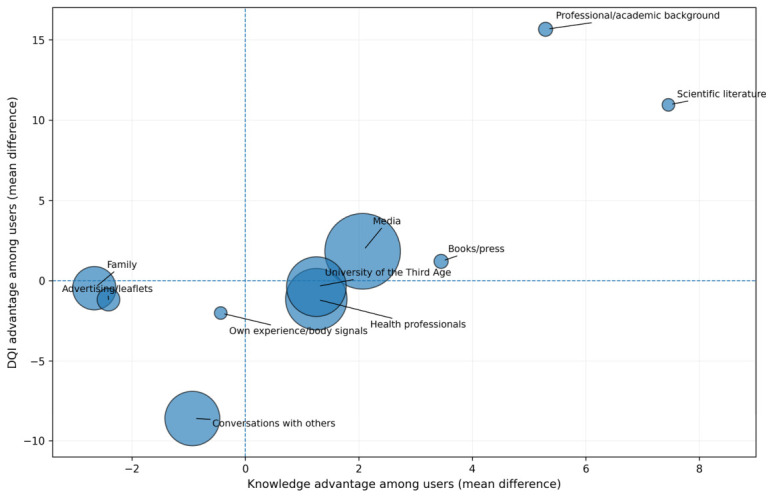
Health-information sources: knowledge and DQI landscape. The figure shows the position of different sources of health information in relation to nutrition knowledge and Diet Quality Index (DQI). Positive values indicate higher knowledge or DQI among users of a given source compared with non-users, whereas negative values indicate lower values.

Health-information sources differed both in frequency of use and in their relationship with nutrition knowledge and DQI (Figure 4; Table S8). Media, health professionals, the University of the Third Age, and conversations with others were the most frequently indicated sources. The most favorable position in the knowledge–diet quality landscape was observed for respondents indicating a professional or academic background and scientific literature as information sources. Health professionals and U3A were associated with higher knowledge scores, but not with a corresponding advantage in DQI, whereas conversations with others and family sources were positioned less favorably.

In the psychosocial domain, self-care and recovery behaviors were mainly activity- and socially oriented (Table S3). Physical and mental activity, social contact, regular health check-ups, social meetings, and cognitive or physical leisure activities were reported most frequently, whereas stress reduction, avoidance of unhealthy snacks, and limited opportunities for rest were less commonly indicated.

### 3.7. Correlations, Group Comparisons, and Association Models

**Figure 5:**
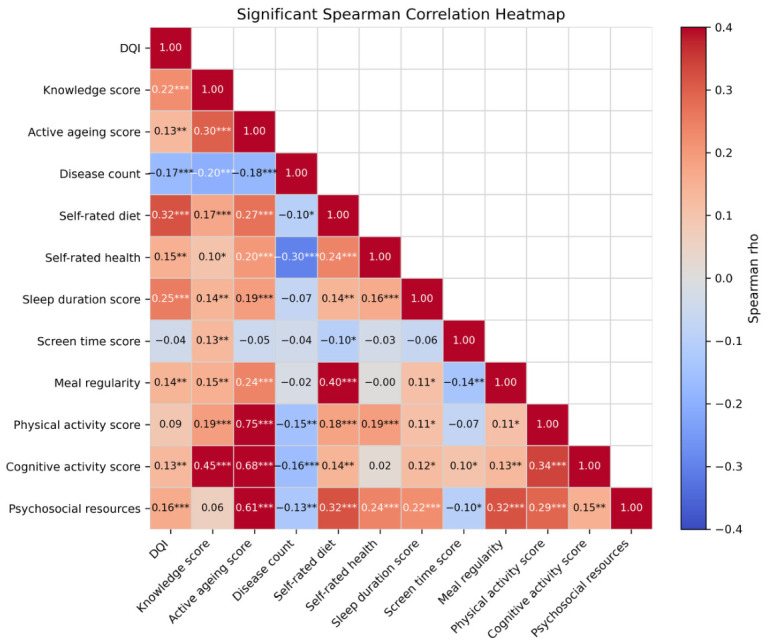
FDR-corrected significant Spearman correlation heatmap. The heatmap presents Spearman correlation coefficients after Benjamini–Hochberg false discovery rate correction. Positive correlations indicate that higher values of one variable are associated with higher values of another variable, whereas negative correlations indicate inverse relationships. Asterisks indicate levels of statistical significance: * *p* < 0.05, ** *p* < 0.01, *** *p* < 0.001.

After Benjamini–Hochberg false discovery rate correction, higher DQI was positively associated with self-rated diet, nutrition knowledge, sleep duration, physical activity, psychosocial resources, self-rated health, meal regularity, and cognitive activity, whereas disease count and stress-related eating were negatively associated with DQI (Figure 5; Table S10). Age, number of meals per day, BMI, screen time, and waist circumference were not significantly associated with DQI after FDR correction.

Non-parametric group comparisons showed higher DQI among women, participants with higher education, and respondents reporting regular meal timing, whereas lower DQI was observed among participants with multimorbidity (Tables S6A and S11A). Multi-group comparisons indicated significant differences in DQI across knowledge categories, active-ageing tertiles, meal-regularity categories, and stress-related eating categories, with the strongest contrasts observed for nutrition knowledge and active-ageing level (Tables S6B and S11B).

In the adjusted models, female sex and sleep score were positively associated with continuous DQI, whereas disease count was inversely associated. In the logistic model for upper-tertile DQI, female sex, older age, higher nutrition knowledge, physical activity, and sleep score were associated with greater odds of higher diet quality, while disease count was associated with lower odds (Figure S6; Table S11). Supplementary exploratory models showed that abdominal obesity was positively associated with female sex, disease count, and nutrition knowledge, and inversely associated with physical activity, whereas multimorbidity was positively associated with age and inversely associated with DQI (Figures S7 and S8). Diagnostic assessment of the adjusted OLS model is provided in Table S12.

### 3.8. Interaction Analyses and Profiles of the Studied Seniors

Interaction analysis showed that the predicted probability of achieving a healthy diet-quality category increased with higher nutrition knowledge in both physical-activity strata (Figure S9). Across the knowledge-score range, this probability remained higher among participants with greater physical activity, suggesting a combined favorable association of knowledge and activity with healthier diet quality.

Profile segmentation identified two distinct profiles within the U3A cohort (Table S13; Figure S10). The health-engaged/higher-resource profile comprised the majority of participants and was characterized by a more favorable overall pattern of diet quality, nutrition knowledge, active-ageing resources, and lower morbidity burden. In contrast, the lower-resource/nutritionally vulnerable profile represented a smaller but clearly distinguishable subgroup with lower nutritional and psychosocial resources and a higher burden of chronic disease. The segmentation heatmap confirmed a consistent contrast between the two profiles across nutritional, lifestyle, psychosocial, and health-related dimensions (Figure S10).

The supplementary model for supplement use showed that female sex and higher nutrition knowledge were positively associated with supplement use, whereas age, higher education, disease count, and DQI were not significant predictors in the adjusted model (Figure S11).

## 4. Discussion

The present study provides a multidimensional assessment of diet quality, nutrition knowledge, lifestyle, and health-related characteristics among older adults attending Universities of the Third Age in Poland. This approach corresponds with the concept of healthy ageing, which emphasizes not only longer survival but also the maintenance of functional ability, well-being, and quality of life in later life [21,22]. In this context, dietary patterns and lifestyle behaviours represent important modifiable factors that may support functional capacity and reduce the burden of diet-related chronic conditions in older populations [23,24]. Therefore, identifying dietary patterns, food preferences, and nutrition-related competencies may help target nutrition education more precisely and improve the relevance of health-promotion activities for older adults [25]. Overall, the studied U3A population was characterized by moderate diet quality and a relatively favorable lifestyle profile, including regular meal consumption and moderate social and cognitive activity. At the same time, substantial variation in nutrition knowledge and clear gaps in more detailed nutritional issues were observed, which is consistent with the findings of Kucharska et al. [26], who reported an uneven distribution of nutrition-related competencies in this population group.

The predominance of neutral diet quality in the studied U3A population indicates that dietary habits were not clearly unfavorable, but also did not fully correspond to an optimal health-promoting dietary model. This suggests a discrepancy between relatively moderate nutrition knowledge and its practical translation into daily food choices. Similar findings have been reported in Polish older adults, where nutritional ambivalence and limited adherence to dietary recommendations remain common [27,28]. In the present study, higher DQI was driven mainly by fruit, vegetables, wholemeal bread, and fermented dairy products, which is consistent with dietary recommendations for older adults [29]. These foods may also support gut microbiota diversity, which is increasingly recognized as relevant to inflammation, metabolic regulation, cognitive function, and frailty risk during ageing [30,31]. Conversely, lower DQI was associated mainly with butter, refined bread and bakery products, processed meat, sweets, and yellow cheese, reflecting dietary choices rich in saturated fat, refined carbohydrates, and processed products. This corresponds with Polish dietary traditions characterized by a relatively high preference for animal fats and processed meat products, which have been associated with unfavorable cardiometabolic profiles and increased chronic disease risk [32,33,34,35].

Declared culinary practices provided an additional context for interpreting diet quality. The predominance of boiling, steaming, stewing, and raw or minimally processed foods, together with the rare use of deep-fat frying and grilling, indicates a generally favorable food-preparation profile and potentially lower exposure to compounds formed during high-temperature meat processing, such as heterocyclic amines and polycyclic aromatic hydrocarbons [36,37]. This profile may partly reflect participation in U3A educational activities, as nutrition education can support more conscious dietary and culinary choices among older adults [38,39]. At the same time, the preference for natural honey and sugar, with rare use of low-calorie sweeteners, is consistent with earlier observations that older or chronically ill adults may be cautious toward non-sugar sweeteners [40].

The PCA-derived dietary patterns further confirmed the heterogeneity of dietary behaviors in the studied group. The three retained patterns—convenience/ultra-processed, prudent/health-promoting, and traditional meat-and-fat—were conceptually consistent with dietary-pattern typologies previously described in Polish older adults [33,41]. The convenience/ultra-processed pattern reflected higher consumption of sugar-sweetened beverages, fast food, highly processed products, and lard, corresponding broadly to ultra-processed food consumption, which has been linked to higher cardiometabolic and chronic disease risk [41,42,43]. In contrast, the prudent/health-promoting pattern included wholegrain products, legumes, fish, white meat, and fermented dairy products, resembling dietary structures such as Mediterranean or DASH-type patterns that are associated with more favorable cardiovascular and multimorbidity-related outcomes [44,45,46,47,48]. The traditional meat-and-fat pattern, characterized by processed meat, white bread, red meat, butter, and yellow cheese, reflected Central and Eastern European dietary traditions and may require targeted educational strategies focused on reducing saturated fat and processed meat intake while preserving culturally acceptable food choices [27].

Żarnowski et al. [49], based on a representative cross-sectional study of adults, including 248 respondents aged 60 years and older, reached corresponding conclusions indicating the existence of a substantial nutrition knowledge gap among Polish adults [49]. In the present study, the mean nutrition knowledge score was 11.34 ± 4.93 points on a 0–24 scale, indicating a moderate level of nutrition-related competence. This result is consistent with the findings of Kucharska et al. [26], who used the KomPAN questionnaire [26,50].

The applied scale demonstrated very good psychometric properties, as confirmed by a high Cronbach’s alpha value of 0.856. The Kaiser–Meyer–Olkin (KMO) measure reached 0.855, indicating the suitability of the scale for population-based assessment of nutrition knowledge among older adults. Analysis of the response structure revealed differences in the difficulty level of individual items. Respondents demonstrated relatively good knowledge of basic dietary principles related to, among others, the role of vitamin D, dietary fiber, fermented dairy products, and salt reduction. However, clear knowledge gaps were identified in more complex areas, such as mineral balance, specific micronutrient deficiencies, and more detailed dietary recommendations.

These knowledge gaps are of considerable importance because they are directly related to the prevention and management of diseases commonly occurring in older adults, including cardiovascular diseases and osteoporosis. The present study confirmed a positive association between nutrition knowledge and a higher probability of achieving DQI values in the upper tertile in a logistic model adjusted for covariates. This is consistent with studies indicating an important, although complex, role of nutrition-related competencies as determinants of dietary behaviors [51]. An unexpected finding of the supplementary abdominal-obesity model was the positive association between nutrition knowledge and abdominal obesity. This result should be interpreted with caution and does not imply that greater nutrition knowledge increases the risk of abdominal obesity. Given the cross-sectional design, this association may reflect reverse causality: individuals with abdominal obesity may have had greater previous exposure to medical advice, dietary counselling, weight-control recommendations, or health-promotion messages, which could increase nutrition knowledge without necessarily translating into sustained behavioural change. Residual confounding related to previous diagnoses, medication use, duration of obesity, motivation to change, or prior participation in lifestyle interventions also cannot be excluded. Moreover, abdominal obesity was defined using self-reported waist circumference, which may be affected by measurement error. Therefore, this supplementary finding should be regarded as hypothesis-generating and requires confirmation in longitudinal studies using objective anthropometric assessment.

Physical activity was positively related to diet quality in the correlation analysis and was associated with higher odds of achieving upper-tertile DQI in the adjusted logistic model, although its association with continuous DQI was attenuated and no longer statistically significant after applying HC3 robust standard errors in the OLS model. This finding is consistent with the well-documented clustering of health behaviors, whereby physical activity and healthier dietary choices co-occur as components of a health-promoting lifestyle. Xu et al. [52] showed that, in older populations, higher diet quality and greater physical activity, although independent, jointly contribute to a more favorable health profile and better self-rated health [52]. This corresponds with the findings of the present study regarding the positive relationship between nutrition knowledge and active ageing, including physical and cognitive activity.

The favorable association observed in the present study between sleep profile and higher DQI is consistent with the systematic review by Godos et al. [53], which included 29 studies analyzing the relationship between sleep quality and diet. That review showed that higher intake of foods characteristic of healthy dietary patterns, such as vegetables, fruit, and wholegrain products, was associated with more favorable sleep parameters, whereas unhealthy dietary patterns correlated with poorer sleep quality [53,54]. Insomnia represents an important health problem in older adults and is a risk factor for reduced quality of life, mental disorders, and cardiovascular diseases [55].

The analysis of the studied cohort showed that multimorbidity was significantly associated with lower diet quality compared with respondents who declared no chronic diseases or only one condition. Moreover, a higher number of disease entities was associated with lower DQI values in both linear and logistic models. These findings are consistent with prospective studies in older populations, where higher diet quality was associated with fewer chronic diseases and a slower rate of multimorbidity development [56]. They also correspond with the cohort study by Abbad-Gomez et al. [57], which suggested that adherence to healthy dietary patterns may significantly modulate the pace of multimorbidity development in older adults. Diets such as MIND, AHEI, and AMED were associated with slower accumulation of chronic diseases, whereas a diet with higher inflammatory potential was related to a less favorable health profile [57].

The most frequently reported barrier to following healthy eating principles among respondents was long-established dietary habits. Additional barriers included insufficient motivation, financial limitations, and/or deficits in nutrition knowledge. These findings are consistent with the meta-analysis by Walker-Clarke et al. [58], which indicated that behaviors shaped over many years are highly persistent and relatively resistant to change, even when nutrition knowledge is adequate. This is because everyday dietary choices in older adults are influenced by individual factors, motivation, and the broader environment [58,59].

The findings of the present study also correspond with the systematic review by Teggart et al. [60], which showed that group-based nutrition interventions among community-dwelling older adults are more promising when nutrition education is combined with behavior-change techniques, goal setting, action planning, practical exercises, tastings, and/or culinary demonstrations [60]. In relation to U3A activities, the present findings suggest that effective nutrition education programs should include not only the transfer of nutrition knowledge but also elements supporting the development of practical skills, strengthening motivation, and consolidating health-promoting dietary patterns among older adults. This is consistent with earlier research findings [60].

The analysis of respondents allowed the identification of two distinct profiles of U3A participants, differing in health status, diet quality, and psychosocial functioning. The first profile comprised younger older adults with higher educational attainment and was characterized by more favorable diet quality, higher nutrition knowledge, greater physical and cognitive activity, and lower multimorbidity burden. The second profile included older participants with lower educational attainment and was characterized by poorer diet quality, lower activity levels, and a greater burden of chronic diseases.

Importantly, despite participation in the same form of senior education, namely U3A, differences between the profiles were observed, demonstrating the heterogeneity of the studied population of U3A participants. These findings challenge the common perception of U3A participants as a relatively homogeneous group in terms of health resources, competencies, and health-promoting behaviors. From the perspective of public health, clinical practice, and dietetics, the identification of a subgroup of older adults more vulnerable to unfavorable dietary patterns and more heavily burdened by chronic diseases is of particular importance. The identification of the lower-resource/nutritionally vulnerable profile has direct practical implications for health-promotion activities within Universities of the Third Age. This subgroup may require a different educational approach than participants who are already health-engaged and resource-rich. Instead of relying solely on general lectures on healthy eating, interventions for this group should prioritize practical, low-threshold strategies, including simple meal-planning guidance, affordable healthy food options, support in translating nutrition knowledge into daily routines, and repeated reinforcement of key messages. Because this profile was also characterized by lower activity resources and a greater morbidity burden, nutrition education should be integrated with physical-activity promotion, chronic-disease self-management, and social-support components. Such targeted programs may help reduce inequalities within the U3A population itself and increase the effectiveness of healthy-ageing interventions among participants with the greatest need for support.

Ter Borg et al. [61] indicated a high risk of nutrient inadequacies among older adults, which may contribute to frailty, falls, cognitive decline, and multimorbidity [61]. In this context, the socially and cognitively active lifestyle observed among many U3A participants should be considered a protective resource, as stronger social relationships are associated with better long-term health outcomes and lower mortality risk [62,63]. However, the coexistence of a lower-resource/nutritionally vulnerable subgroup indicates that U3A-based interventions should combine nutrition education with social support, activity promotion, and chronic disease self-management components.

## 5. Study Limitations

The present study has several limitations that should be considered when interpreting the findings. First, due to its cross-sectional design, causal relationships between nutrition knowledge, diet quality, lifestyle factors, and health outcomes cannot be inferred. Second, the data were collected using a self-administered online questionnaire, which may have introduced recall bias and social desirability bias, particularly in relation to dietary behaviors, physical activity, anthropometric measures, and self-reported diseases. Third, the study population consisted exclusively of older adults attending Universities of the Third Age, which represents a highly selected subgroup of the older population. The sample was predominantly female, highly educated, largely urban, and characterized by relatively high levels of social and cognitive activity. These characteristics may be associated with greater health awareness, better access to information, and stronger engagement in health-promoting behaviors than would be expected in the broader older population. Therefore, the findings should not be interpreted as representative of all older adults in Poland, particularly those with lower educational attainment, limited social participation, rural residence, poorer digital access, or greater functional limitations. Fourth, the PCA-derived dietary-pattern solution explained 38.6% of the total variance in food-consumption frequency, indicating that the retained patterns captured only part of the complexity of dietary behaviour in this population. Therefore, these patterns should be interpreted as broad, exploratory dietary profiles rather than exhaustive representations of all eating behaviours among U3A participants.

Despite these limitations, the study provides valuable insight into the multidimensional relationships between nutrition knowledge, diet quality, lifestyle, and health status in a specific and highly relevant group of community-dwelling older adults. The relatively large analytical sample, use of established diet-quality indicators, psychometric evaluation of the knowledge scale, and integration of multivariable, pattern-based, and segmentation analyses strengthen the interpretative value of the findings.

## 6. Conclusions

The findings of the present study indicate that older adults attending Universities of the Third Age in Poland constitute an educationally and socially active, but heterogeneous, population in terms of diet quality, nutrition knowledge, lifestyle, and health burden. The predominance of a neutral diet-quality profile suggests that the respondents’ dietary patterns were not clearly unfavorable, yet they did not fully correspond to the most beneficial model of nutrition in the context of healthy ageing.

A more favorable dietary profile was associated with higher nutrition knowledge and a healthier lifestyle, including greater physical activity, better sleep quality, and more regular meal consumption. At the same time, multimorbidity and a greater chronic disease burden were linked to lower diet quality, emphasizing the need to include dietary assessment and nutritional counseling in comprehensive care for older adults, particularly those affected by chronic conditions.

The study also revealed important gaps in nutrition knowledge, especially in more complex areas such as mineral balance, micronutrient deficiencies, and detailed dietary recommendations. These findings suggest that nutrition education for older adults attending U3A should go beyond general dietary advice and should be more precisely targeted, practical, and behavior-oriented.

These findings should be interpreted in relation to the specific characteristics of the study population. The sample consisted of older adults attending Universities of the Third Age in Poland, predominantly women and individuals with relatively high educational attainment and high levels of social and cognitive activity. Therefore, the results should not be generalized to the entire older population. Rather, they provide evidence for designing targeted, practical, and behaviour-oriented nutrition-education strategies within U3A and similar educational settings for socially active older adults. Particular attention should be paid to participants with lower health-related resources, poorer diet quality, and a higher burden of multimorbidity, as they may benefit most from individualized and sustained educational interventions.

## Figures and Tables

**Table 1:** Sociodemographic, anthropometric, and clinical characteristics of the study population.

Domain	Category/Statistic	Value *
Sample size	Total sample	450
Age	Mean ± SD, years	73.63 ± 5.73; 73 [70–77]
Sex	Female	373 (82.9%)
	Male	77 (17.1%)
Age group	60–69 years	89 (19.8%)
	70–74 years	187 (41.6%)
	75+ years	174 (38.7%)
Residence	City > 500,000 inhabitants	259 (57.6%)
	City 100,000–500,000 inhabitants	46 (10.2%)
	City 10,000–100,000 inhabitants	89 (19.8%)
	Town < 10,000 inhabitants	30 (6.7%)
	Rural area	26 (5.8%)
Education	Higher education	265 (58.9%)
	Secondary education	109 (24.2%)
	Vocational education	44 (9.8%)
	Higher education with doctoral degree	22 (4.9%)
	Post-secondary education	4 (0.9%)
	Higher education with professor title	2 (0.4%)
	Two-year post-secondary college	2 (0.4%)
	Higher/postgraduate education	2 (0.4%)
Anthropometry	Height, m; Mean ± SD	1.64 ± 0.07; 1.64 [1.60–1.68]
	Weight, kg; Mean ± SD	70.31 ± 11.71; 70.0 [62.0–78.0]
	BMI, kg/m^2^, Mean ± SD	26.05 ± 4.16; 25.46 [23.56–28.34]
	Waist circumference, cm; Mean ± SD	87.42 ± 13.65; 86.0 [78.0–95.0]
BMI category	Underweight	8 (1.8%)
	Normal weight	196 (43.6%)
	Overweight	175 (38.9%)
	Obesity	71 (15.8%)
Abdominal obesity	Yes	186 (41.3%)
	No	260 (57.8%)
Self-rated health	Very good	28 (6.2%)
	Good	233 (51.8%)
	Average	167 (37.1%)
	Poor	20 (4.4%)
	Very poor	2 (0.4%)
Self-rated physical activity	Low (mostly sedentary)	80 (17.8%)
	Moderate	316 (70.2%)
	High	48 (10.7%)
	None	6 (1.3%)
Declared disease burden	Disease count; mean ± SD	1.12 ± 0.66
	Multimorbidity	62 (13.8%)

* Continuous variables are presented primarily as medians [IQR] because of non-normal or skewed distributions; mean ± SD values are additionally reported for comparability. Categorical variables are shown as *n* (%). One implausible waist-circumference value was treated as missing.

**Table 2:** Diet quality indices and category distribution.

Domain	Category/Statistic *	Value	Additional
Diet quality indices	pHDI (points)	28.40 [19.90–36.40]	29.15 ± 13.78
	nHDI (points)	12.21 [6.23–19.34]	13.95 ± 9.79
	DQI (points)	15.59 [3.93–24.86]	15.19 ± 16.33
DQI 3-category	Healthy	108	24.0%
	Neutral	340	75.6%
	Unhealthy	2	0.4%

* DQI = Diet Quality Index; pHDI = pro-Healthy Diet Index; nHDI = non-Healthy Diet Index. Diet-quality indices are presented as medians [IQR] because of non-normal distributions; mean ± SD values are additionally reported for comparability.

**Table 3:** Interpretation of PCA-derived dietary patterns based on dominant loadings.

Pattern	Eigenvalue	Explained Variance (%)	Cumulative Variance (%)	Dominant Food Groups/Highest Loadings	Interpretation
Pattern 1	4.268	17.04	17.04	Energy drinks (0.716); sugar-sweetened beverages (0.684); canned meat (0.644); lard (0.621); fast food (0.614)	Convenience/ultra-processed pattern
Pattern 2	3.280	13.09	30.13	Whole grains/groats (0.661); fish (0.647); white meat (0.632); fermented dairy (0.553); legumes (0.552)	Prudent/health-promoting pattern
Pattern 3	2.127	8.49	38.62	Processed meat (0.738); white bread (0.669); red meat (0.584); butter (0.571); yellow cheese (0.571)	Traditional meat-and-fat pattern

Components were retained based on eigenvalues >1.0, scree plot inspection, explained variance, and interpretability. Varimax rotation was applied. Food groups were assigned to components based on absolute rotated loadings ≥0.30. Full rotated component loadings are provided in Table S9.

**Table 4:** Nutrition knowledge profile and psychometric properties of the knowledge scale.

Domain	Indicator	Value	Additional
Knowledge performance	Knowledge score, points	12.00 [9.00–15.00]	11.34 ± 4.93
	Number of items	24	Single-score summed scale
Psychometric properties	Cronbach’s alpha	0.856	Very good internal consistency
	KMO	0.855	Very good sampling adequacy
	Bartlett’s χ^2^	2749.10	*df* = 276; *p* < 0.001
Item diagnostics	Observed item-total rho range	0.159–0.520	Based on item-level diagnostics
	Observed alpha-if-deleted range	0.844–0.857	No single item materially improved scale reliability
Best-performing item	Sun exposure promotes vitamin D synthesis in the body	87.6% correct	11.1% “do not know”
Most difficult item	In a proper diet, the calcium-to-phosphorus ratio should be 1:1	4.0% correct	83.1% “do not know”

The nutrition knowledge score is presented as median [IQR] because of its non-normal distribution; mean ± SD is additionally reported for comparability. KMO = Kaiser–Meyer–Olkin measure.

## Data Availability

The original contributions presented in this study are included in the article/Supplementary Material. Further inquiries can be directed to the corresponding author.

## Supplementary Materials

The following supporting information can be downloaded at: https://www.mdpi.com/article/10.3390/nu18122025/s1, Table S1. Construction and theoretical ranges of composite lifestyle and psychosocial scores; Table S2: Health status, lifestyle, and core dietary behaviors; Table S3: Self-care and rest behaviours; Table S4A: Sweetening, snack choices, and cooking practices: Snack/between-meal choices; Table S4B. Sweetening, snack choices, and cooking practices: Cooking practices; Table S4C. Sweetening, snack choices, and cooking practices: Sweetening choices; Table S5: Detailed supplement profile; Table S6A. Extended non-parametric binary-group DQI comparisons with effect sizes; Table S6B. Post hoc pairwise comparisons following Kruskal–Wallis tests for DQI; Table S7. Item diagnostics and knowledge gaps for the nutrition knowledge scale; Table S8. Exact values for the health-information source landscape; Table S9. Full rotated PCA loading matrix for food-frequency variables; Table S10. Spearman correlation matrix with raw *p* values and FDR-adjusted q values; Table S11. Group comparisons and multivariable associations for diet quality; Table S12. Diagnostic assessment of the adjusted OLS model; Table S13. Summary of identified senior profiles; Figure S1. Disease co-occurrence network; Figure S2. PCA scree plot; Figure S3: PCA loadings heatmap; Figure S4: Diet clusters in PCA space; Figure S5: Barriers to healthy eating; Figure S6: Predictors of higher diet quality (forest plot); Figure S7: Predictors of abdominal obesity; Figure S8: Predictors of multimorbidity; Figure S9: Predicted probability of healthy diet quality by nutrition knowledge and physical activity; Figure S10: Senior profile segmentation heatmap; Figure S11: Predictors of supplement use.
