# Supplementary material for: A Cross-Sectional Study of Nutrition Knowledge, Diet Quality, Lifestyle, and Health Profiles Among Older Adults Attending Universities of the Third Age in Poland
Source: Nutrients. 2026 Jun 22;18(12):2025. doi: 10.3390/nu18122025 (PMC13305659; doi:10.3390/nu18122025)
Supplement: Supplementary file 1 [file nutrients-18-02025-s001.zip › nutrients-4357456-supplementary.pdf]

Table S1. Construction and theoretical ranges of composite lifestyle and psychosocial scores.

Composite score	Source variables/items	Coding and construction	Theoretical range	Interpretation
Physical activity score	Frequency of selected physical activities: walking/marching, swimming, Nordic walking, cycling, gymnastics, and dancing.	Each activity-frequency item was coded on an ordinal scale: never = 0, less than once per month = 1, once per month = 2, once every two weeks = 3, once per week = 4, and daily = 5. The score was calculated as the sum of the six activity items.	0–30 points	Higher values indicate more frequent engagement in physical activity.
Cognitive activity score	Frequency of cognitively stimulating activities: language learning, participation in studies/educational activities, courses or workshops, chess, card games, strategic games, crosswords/Sudoku, and reading.	Each activity-frequency item was coded on the same ordinal scale: never = 0, less than once per month = 1, once per month = 2, once every two weeks = 3, once per week = 4, and daily = 5. The score was calculated as the sum of the eight cognitive-activity items.	0–40 points	Higher values indicate greater engagement in cognitively stimulating activities.
Sleep score	Self-reported average sleep duration per day.	Sleep duration was recoded to reflect a more favorable sleep profile. Sleeping 7–8 h/day was assigned the highest score, whereas shorter or longer sleep duration was assigned a lower score.	1–2 points	Higher values indicate a more favorable sleep-duration profile.
Meal regularity score	Self-reported regularity of daily meal timing.	Responses were coded as: no regular meal timing = 0, sometimes regular meal timing = 1, and regular meal timing = 2.	0–2 points	Higher values indicate more regular daily meal timing.
Psychosocial resources score	Items reflecting psychosocial and personal resources, including perceived ability to cope with stress, sense of safety, perceived self-efficacy and control, adaptability to new circumstances, self-worth, attentiveness to one's own feelings and needs, and availability of social contacts/support.	Responses were recoded so that higher values reflected more favorable psychosocial resources. Because the source items had different response formats, each item was transformed to a 0–100 scale and the final score was calculated as the mean of the recoded items.	0–100 points	Higher values indicate greater psychosocial resources and perceived support.
Active ageing score	Integrated indicator combining physical activity, cognitive activity, psychosocial resources, sleep, and meal regularity domains.	Component scores were standardized as z-scores and then averaged to obtain a global active-ageing score.	No fixed theoretical range because the score is standardized; values are centered around 0 in the analytical sample.	Higher positive values indicate a more favorable active-ageing profile, whereas lower negative values indicate lower lifestyle and psychosocial resources.

Table S2. Health status, lifestyle, and core dietary behaviors.

Domain	Category / statistic	Value
**Daily lifestyle**	Sleep duration: <=6 h	142 (31.6%)
	Sleep duration: 7-8 h	281 (62.4%)
	Sleep duration: >=9 h	27 (6.0%)
	Meals per day: 2	18 (4.0%)
	Meals per day: 3	231 (51.3%)
	Meals per day: 4	150 (33.3%)
	Meals per day: 5	51 (11.3%)
	Regular meal timing: yes	276 (61.3%)
	Regular meal timing: sometimes	109 (24.2%)
	Regular meal timing: no	65 (14.4%)
**Self-care behaviours**	Physically and mentally active	287 (63.8%)
	Meeting other people for well-being	218 (48.4%)
	Regular health check-ups	182 (40.4%)
	Regular medication intake	145 (32.2%)
	Avoiding overeating	80 (17.8%)
	Avoiding sweets and unhealthy snacks	56 (12.4%)
**Rest and leisure**	Social meetings	268 (59.6%)
	Physical activity	243 (54.0%)
	Mental activity	235 (52.2%)
	Travelling / sightseeing	184 (40.9%)
	Sleep / passive rest	156 (34.7%)
**Snacks between meals**	Fruit	332 (73.8%)
	Vegetables	257 (57.1%)
	Nuts / seeds	163 (36.2%)
	Sweet snacks	128 (28.4%)
	Salty snacks	2 (0.4%)
	Sweetened dairy desserts/drinks	22 (4.9%)
**Cooking methods**	Boiling / steaming	290 (64.4%)
	Raw / no heat treatment	225 (50.0%)
	Stewing	225 (50.0%)
	Baking	133 (29.6%)
	Shallow frying	124 (27.6%)
	Blanching	80 (17.8%)
	Grilling	16 (3.6%)
	Deep frying	2 (0.4%)
**Sweetening choices**	Natural honey	179 (39.8%)
	White sugar	119 (26.4%)
	Brown / cane sugar	80 (17.8%)
	Polyol sweeteners	30 (6.7%)
	Classic sweeteners	12 (2.7%)
**Supplement use**	Any supplement use	261 (58.0%)
	Vitamin D	107 (23.8%)
	Magnesium	105 (23.3%)
	Omega-3 / fish oil	37 (8.2%)
	B vitamins	34 (7.6%)

Values are shown as n (%). Snack rows indicate daily or more frequent consumption. Cooking-method rows indicate use at least several times per week.

Table S3. Self-care and rest behaviours.

A: Self-care behaviour	N	Percent
Physically and mentally active	287	63.8
Social contact that improves well-being	218	48.4
Regular health check-ups	182	40.4
Regular medication use	145	32.2
Avoid overeating	80	17.8
Avoid sweets and unhealthy snacks	56	12.4
Physically active	55	12.2
Use dietary supplements	54	12.0
Follow an easily digestible diet	50	11.1
Avoid stimulants	46	10.2
Attend to my own needs	42	9.3
Mentally active	38	8.4
Get sufficient sleep	27	6.0
Try to avoid stress	6	1.3
Other individual responses	4	0.9
**B: Rest and recovery modes**		
Meeting with other people	268	59.6
Physical activity	243	54.0
Cognitive / mental activity	235	52.2
Travelling / sightseeing	184	40.9
Sleeping	156	34.7
Other individual responses	36	8.0
Insufficient time for rest - caring for family members	16	3.6
Insufficient time for rest - still professionally active	14	3.1

Because these were multi-response items. percentages do not sum to 100%.

Table S4A. Sweetening. snack choices. and cooking practices: Snack / between-meal choices.

Snack / between-meal item	Several times/day	Once/day	Several times/week	Once/week	1-3 times/month	Never
Fruit	21.1	52.7	15.6	3.1	2.7	4.9
Vegetables	20.7	36.4	23.6	6.0	2.7	10.7
Sweet snacks	7.1	21.3	21.3	16.9	14.0	19.3
Salty snacks	0.0	0.4	7.6	8.4	20.0	63.6
Sweetened beverages / dairy desserts	1.3	3.6	8.9	6.7	19.1	60.4
Nuts / seeds	10.9	25.3	29.3	7.6	12.7	14.2

Table S4B. Sweetening. snack choices. and cooking practices: Cooking practices (percent of total sample).

Cooking practice	Several times/day	Once/day	Several times/week	Once/week	1-3 times/month	Never
Raw / no thermal processing	12.0	21.1	16.9	5.8	9.8	34.4
Blanching	0.4	3.1	14.2	12.0	19.6	50.7
Boiling / steaming	4.0	25.3	35.1	12.0	12.9	10.7
Stewing	2.2	6.7	41.1	19.6	19.1	11.3
Baking	2.2	3.1	24.2	23.6	28.9	18.0
Grilling	0.0	1.3	2.2	3.1	25.6	67.8
Low-fat frying	1.3	4.4	21.8	18.2	30.9	23.3
Deep-fat frying	0.0	0.4	0.0	1.8	13.8	84.0

Frequency categories are mutually exclusive. Values in Panels B and C are percentages of the full sample (N = 450).

Table S4C. Sweetening. snack choices. and cooking practices: Sweetening choices.

Sweetening option	N	Percent of total sample
Natural honey	179	39.8
White sugar	119	26.4
Brown / cane sugar	80	17.8
Polyol sweeteners (xylitol/erythritol etc.)	30	6.7
Artificial sweeteners	12	2.7
Artificial honey	4	0.9
Coconut sugar	2	0.4

Teaspoon amount among respondents who reported sweetening beverages or foods

Teaspoon amount	N	Percent among respondents who sweeten	Percent of total sample
1 teaspoon	189	77.5	42.0
2 teaspoons	41	16.8	9.1
3 teaspoons	8	3.3	1.8
4 teaspoons	4	1.6	0.9
5 teaspoons or more	2	0.8	0.4

Table S5. Detailed supplement profile.

Supplement category	N	Percent of total sample	Percent of supplement users
Vitamin D	107	23.8	41.0
Magnesium	105	23.3	40.2
Omega-3/fish oil	37	8.2	14.2
B vitamins	34	7.6	13.0
Lutein	18	4.0	6.9
Zinc	12	2.7	4.6
Calcium	12	2.7	4.6
Vitamin C	10	2.2	3.8
Iron	4	0.9	1.5
Multivitamin	2	0.4	0.8

Overall supplement use was reported by 261 respondents (58.0%). The most common categories were vitamin D and magnesium.

Table S6A. Extended non-parametric binary-group DQI comparisons with effect sizes.

Comparison	Group 1 n	Group 1 DQI median [IQR]	Group 0 n	Group 0 DQI median [IQR]	Mann–Whitney U	Rank-biserial r	p	q-FDR
Female vs male	373	16.17 [5.43–24.99]	77	10.97 [−2.99–21.19]	17108.5	0.191	0.008	0.017
Age ≥75 vs <75	174	12.84 [3.99–24.13]	276	15.97 [3.11–25.66]	22570.5	−0.060	0.283	0.331
Higher education vs other	291	16.86 [5.97–25.66]	159	11.43 [2.32–21.87]	26548.0	0.148	0.010	0.017
Abdominal obesity vs no	186	13.87 [5.36–24.14]	260	15.81 [0.04–26.01]	24166.5	−0.001	0.992	0.992
Multimorbidity vs none/one disease	62	9.89 [−1.30–16.02]	388	16.32 [5.42–25.84]	8217.0	−0.317	<0.001	<0.001
Supplement use vs none	261	16.17 [7.94–24.86]	189	13.94 [0.00–25.71]	27135.0	0.100	0.070	0.098
Regular meals vs irregular/sometimes	276	16.56 [8.20–26.84]	174	11.44 [0.04–22.52]	28084.0	0.170	0.002	0.009

Table S6B. Post hoc pairwise comparisons following Kruskal–Wallis tests for DQI.

Factor	Group 1	Group 2	n1	n2	Mann–Whitney U	Rank-biserial r	Raw p value	q-FDR
	Good	Insufficient	52	101	3814.0	0.452	<0.001	<0.001
Knowledge category	Good	Sufficient	52	297	10356.0	0.341	<0.001	<0.001
	Insufficient	Sufficient	101	297	9955.0	−0.336	<0.001	<0.001
	T1 low	T2 mid	151	149	7743.5	−0.312	<0.001	<0.001
Active ageing tertile	T1 low	T3 high	151	150	9896.0	−0.126	0.058	0.088
	T2 mid	T3 high	149	150	12428.0	0.112	0.094	0.094
	No	Sometimes	65	109	3388.0	−0.044	0.632	0.632
Meal regularity	No	Yes	65	276	7432.0	−0.171	0.032	0.047
	Sometimes	Yes	109	276	12508.0	−0.168	0.010	0.030
	No	Sometimes	239	118	16250.0	0.152	0.019	0.057
Stress-related eating	No	Yes	239	93	12435.0	0.119	0.093	0.139
	Sometimes	Yes	118	93	5366.0	−0.022	0.784	0.784

Table S7. Item diagnostics and knowledge gaps for the nutrition knowledge scale.

Rank	Knowledge item	Correct (%)	Do not know (%)	Item-total rho	Alpha if deleted	Gap class
1	The calcium-to-phosphorus ratio in a healthy diet should be 1:1.	4.0	83.1	0.221	0.856	Very high
2	Insufficient vitamin PP intake may cause dermatitis and diarrhea.	18.7	76.9	0.399	0.851	
3	Phosphorus is a component of nervous tissue.	24.0	70.2	0.396	0.851	
4	Vegetarian diets increase the risk of anemia.	20.4	45.6	0.214	0.857	High
5	Vitamin C-rich fruit improves iron absorption.	49.1	45.6	0.342	0.853	
6	Offal contains considerable amounts of “bad” LDL cholesterol.	16.0	44.0	0.199	0.856	
7	Eating grilled meat promotes cancer risk.	42.7	43.6	0.362	0.852	
8	Complex carbohydrates should be replaced by simple sugars.	36.0	42.7	0.522	0.847	
9	Starting to cook vegetables in cold water preserves nutrients.	42.7	40.0	0.430	0.849	
10	Eating moldy bread can cause Salmonella infection.	19.1	38.7	0.347	0.853	Moderate
11	Yellow cheese is a better calcium source than cottage cheese.	34.0	36.9	0.346	0.853	
12	Protein should be the main source of energy in a healthy diet.	11.1	34.4	0.363	0.852	
13	Butter and fortified margarines are rich in vitamins A and D.	56.4	34.2	0.523	0.846	
14	Cereal products need to be eaten only once daily.	18.7	31.6	0.159	0.857	
15	Fruit and vegetables are sources of “empty calories.”	66.0	26.4	0.399	0.848	
16	Only children and adolescents should drink milk.	60.2	25.1	0.248	0.854	
17	Vegetable oils and olive oil contain a lot of cholesterol.	71.8	23.3	0.503	0.844	
18	Frequent intake of fatty sea fish accelerates atherosclerosis.	72.4	22.7	0.498	0.845	
19	Bio-yogurts contain beneficial intestinal bacteria.	78.0	20.2	0.484	0.846	
20	High salt intake protects against hypertension.	67.3	17.3	0.418	0.848	Low
21	Reducing fatty dishes helps prevent cardiovascular diseases.	76.7	17.3	0.496	0.845	
22	Fruit and/or vegetables should be consumed with every meal.	77.3	16.0	0.325	0.852	
23	Wholemeal bread contains more fiber than refined bread.	83.6	16.0	0.520	0.844	
24	Sun exposure promotes endogenous vitamin D synthesis.	87.6	11.1	0.511	0.846	

Items are ranked by the proportion of "do not know" responses. highlighting the main knowledge gaps.

Table S8. Exact values for the health-information source landscape.

Source	Users_n	Knowledge_delta	DQI_delta	Knowledge_p	DQI_p
Media	289	2.067	1.830	0.000	0.094
Health professionals	191	1.251	-1.152	0.033	0.842
University of the Third Age	180	1.252	-0.372	0.007	0.370
Conversations with others	153	-0.934	-8.598	0.026	0.000
Family	95	-2.662	-0.469	0.000	0.172
Advertising/leaflets	26	-2.412	-1.176	0.058	0.696
Books/press	10	3.450	1.201	0.028	0.833
Professional/academic background	10	5.291	15.647	0.001	0.002
Scientific literature	8	7.456	10.943	0.000	0.017
Own experience/body signals	8	-0.434	-2.013	0.418	0.739

Table S9. Full rotated PCA loading matrix for food-frequency variables.

**Food group**	**Pattern 1**	**Pattern 2**	**Pattern 3**	**max_abs_loading**	**assigned_pattern**	**cross_loading_flag**
Wholemeal bread	-0.027	0.498	-0.193	0.498	Pattern 2	No
Whole grains/groats	0.032	0.661	-0.165	0.661	Pattern 2	No
Milk	0.077	0.433	0.030	0.433	Pattern 2	No
Fermented dairy	-0.127	0.553	-0.102	0.553	Pattern 2	No
Curd cheese	-0.198	0.528	0.281	0.528	Pattern 2	No
White meat	0.090	0.632	0.251	0.632	Pattern 2	No
Fish	0.159	0.647	0.085	0.647	Pattern 2	No
Legumes	0.249	0.552	-0.108	0.552	Pattern 2	No
Eggs	-0.190	0.421	0.277	0.421	Pattern 2	No
Fruit	-0.249	0.408	0.020	0.408	Pattern 2	No
Vegetables	-0.281	0.483	0.021	0.483	Pattern 2	No
White bread	0.059	-0.179	0.669	0.669	Pattern 3	No
White rice/pasta	0.332	0.172	0.498	0.498	Pattern 3	Yes
Fast food	0.614	0.461	0.108	0.614	Pattern 1	Yes
Fried dishes	0.440	0.290	0.294	0.440	Pattern 1	No
Butter	-0.167	0.022	0.571	0.571	Pattern 3	No
Lard	0.621	0.136	0.323	0.621	Pattern 1	Yes
Yellow cheese	0.231	0.224	0.571	0.571	Pattern 3	No
Processed meat	0.040	0.036	0.738	0.738	Pattern 3	No
Red meat	0.243	-0.047	0.584	0.584	Pattern 3	No
Sweets	0.175	0.043	0.477	0.477	Pattern 3	No
Canned meat	0.644	-0.127	0.078	0.644	Pattern 1	No
Sugary soft drinks	0.684	-0.170	0.160	0.684	Pattern 1	No
Energy drinks	0.716	0.003	-0.130	0.716	Pattern 1	No
Alcohol	0.017	0.191	-0.056	0.191	Not assigned (<0.30)	No

Table S10. Spearman correlation matrix with raw p values and FDR-adjusted q values.

variable_1	variable_2	spearman_rho	p_raw	q_fdr	significant_FDR
Age	BMI	0.111	0.018	0.034	TRUE
	Waist circumference	0.165	0.000	0.002	TRUE
	pHDI	0.012	0.807	0.852	FALSE
	nHDI	0.113	0.017	0.032	TRUE
	DQI	-0.061	0.198	0.257	FALSE
	Nutrition knowledge	-0.237	0.000	0.000	TRUE
	Physical activity	0.008	0.870	0.906	FALSE
	Cognitive activity	-0.224	0.000	0.000	TRUE
	Self-rated health	-0.137	0.004	0.009	TRUE
	Self-rated diet	-0.091	0.055	0.085	FALSE
	Screen time	-0.112	0.018	0.034	TRUE
	Sleep duration	-0.108	0.022	0.039	TRUE
	Meals/day	-0.081	0.087	0.121	FALSE
	Meal regularity	-0.065	0.170	0.228	FALSE
	Psychosocial resources	-0.107	0.024	0.041	TRUE
	Disease count	0.216	0.000	0.000	TRUE
	Stress eating	-0.027	0.568	0.634	FALSE
BMI	Waist circumference	0.627	0.000	0.000	TRUE
	pHDI	-0.025	0.598	0.658	FALSE
	nHDI	0.040	0.393	0.466	FALSE
	DQI	-0.051	0.282	0.347	FALSE
	Nutrition knowledge	-0.113	0.016	0.032	TRUE
	Physical activity	-0.195	0.000	0.000	TRUE
	Cognitive activity	-0.083	0.080	0.116	FALSE
	Self-rated health	-0.088	0.061	0.093	FALSE
	Self-rated diet	-0.243	0.000	0.000	TRUE
	Screen time	0.145	0.002	0.005	TRUE
	Sleep duration	-0.046	0.334	0.406	FALSE
	Meals/day	0.030	0.528	0.598	FALSE
	Meal regularity	-0.102	0.030	0.050	FALSE
	Psychosocial resources	-0.105	0.026	0.044	TRUE
	Disease count	0.152	0.001	0.003	TRUE
	Stress eating	0.298	0.000	0.000	TRUE
Waist circumference	pHDI	0.018	0.703	0.749	FALSE
	nHDI	0.004	0.934	0.946	FALSE
	DQI	-0.018	0.705	0.749	FALSE
	Nutrition knowledge	0.137	0.004	0.009	TRUE
	Physical activity	-0.206	0.000	0.000	TRUE
	Cognitive activity	-0.027	0.577	0.639	FALSE
	Self-rated health	-0.054	0.257	0.325	FALSE
	Self-rated diet	-0.144	0.002	0.006	TRUE

	Screen time	0.172	0.000	0.001	TRUE
	Sleep duration	0.003	0.954	0.956	FALSE
	Meals/day	-0.007	0.880	0.909	FALSE
	Meal regularity	-0.005	0.914	0.939	FALSE
	Psychosocial resources	-0.111	0.019	0.035	TRUE
	Disease count	0.037	0.431	0.503	FALSE
	Stress eating	0.237	0.000	0.000	TRUE
pHDI	nHDI	-0.083	0.077	0.112	FALSE
	DQI	0.830	0.000	0.000	TRUE
	Nutrition knowledge	0.199	0.000	0.000	TRUE
	Physical activity	0.171	0.000	0.001	TRUE
	Cognitive activity	0.135	0.004	0.010	TRUE
	Self-rated health	0.088	0.062	0.094	FALSE
	Self-rated diet	0.211	0.000	0.000	TRUE
	Screen time	-0.037	0.431	0.503	FALSE
	Sleep duration	0.221	0.000	0.000	TRUE
	Meals/day	0.114	0.016	0.031	TRUE
	Meal regularity	0.180	0.000	0.001	TRUE
	Psychosocial resources	0.104	0.027	0.046	TRUE
	Disease count	-0.082	0.081	0.116	FALSE
	Stress eating	-0.090	0.057	0.088	FALSE
nHDI	DQI	-0.581	0.000	0.000	TRUE
	Nutrition knowledge	-0.093	0.048	0.077	FALSE
	Physical activity	-0.063	0.186	0.245	FALSE
	Cognitive activity	-0.003	0.956	0.956	FALSE
	Self-rated health	-0.136	0.004	0.009	TRUE
	Self-rated diet	-0.306	0.000	0.000	TRUE
	Screen time	0.063	0.181	0.241	FALSE
	Sleep duration	-0.067	0.153	0.207	FALSE
	Meals/day	0.050	0.295	0.361	FALSE
	Meal regularity	-0.033	0.481	0.555	FALSE
	Psychosocial resources	-0.119	0.012	0.024	TRUE
	Disease count	0.167	0.000	0.001	TRUE
	Stress eating	0.087	0.066	0.098	FALSE
DQI	Nutrition knowledge	0.217	0.000	0.000	TRUE
	Physical activity	0.166	0.000	0.002	TRUE
	Cognitive activity	0.125	0.008	0.017	TRUE
	Self-rated health	0.147	0.002	0.005	TRUE
	Self-rated diet	0.316	0.000	0.000	TRUE
	Screen time	-0.041	0.385	0.461	FALSE
	Sleep duration	0.215	0.000	0.000	TRUE
	Meals/day	0.053	0.259	0.325	FALSE
	Meal regularity	0.141	0.003	0.007	TRUE
	Psychosocial resources	0.163	0.001	0.002	TRUE

	Disease count	-0.172	0.000	0.001	TRUE
	Stress eating	-0.108	0.022	0.039	TRUE
Nutrition knowledge	Physical activity	0.115	0.015	0.029	TRUE
	Cognitive activity	0.315	0.000	0.000	TRUE
	Self-rated health	0.092	0.050	0.080	FALSE
	Self-rated diet	0.164	0.000	0.002	TRUE
	Screen time	0.131	0.005	0.012	TRUE
	Sleep duration	0.218	0.000	0.000	TRUE
	Meals/day	0.158	0.001	0.002	TRUE
	Meal regularity	0.153	0.001	0.003	TRUE
	Psychosocial resources	0.033	0.482	0.555	FALSE
	Disease count	-0.195	0.000	0.000	TRUE
	Stress eating	0.021	0.650	0.710	FALSE
Physical activity	Cognitive activity	0.241	0.000	0.000	TRUE
	Self-rated health	0.212	0.000	0.000	TRUE
	Self-rated diet	0.288	0.000	0.000	TRUE
	Screen time	-0.107	0.023	0.041	TRUE
	Sleep duration	0.133	0.005	0.011	TRUE
	Meals/day	0.031	0.518	0.591	FALSE
	Meal regularity	0.156	0.001	0.003	TRUE
	Psychosocial resources	0.318	0.000	0.000	TRUE
	Disease count	-0.055	0.246	0.313	FALSE
	Stress eating	-0.228	0.000	0.000	TRUE
Cognitive activity	Self-rated health	-0.051	0.281	0.347	FALSE
	Self-rated diet	0.161	0.001	0.002	TRUE
	Screen time	0.094	0.047	0.075	FALSE
	Sleep duration	0.163	0.001	0.002	TRUE
	Meals/day	0.056	0.239	0.307	FALSE
	Meal regularity	0.181	0.000	0.000	TRUE
	Psychosocial resources	0.118	0.012	0.025	TRUE
	Disease count	-0.152	0.001	0.003	TRUE
	Stress eating	-0.127	0.007	0.015	TRUE
Self-rated health	Self-rated diet	0.240	0.000	0.000	TRUE
	Screen time	-0.029	0.546	0.614	FALSE
	Sleep duration	0.157	0.001	0.002	TRUE
	Meals/day	-0.084	0.076	0.112	FALSE
	Meal regularity	-0.004	0.934	0.946	FALSE
	Psychosocial resources	0.243	0.000	0.000	TRUE
	Disease count	-0.303	0.000	0.000	TRUE
	Stress eating	-0.010	0.840	0.880	FALSE
Self-rated diet	Screen time	-0.098	0.037	0.062	FALSE
	Sleep duration	0.190	0.000	0.000	TRUE
	Meals/day	0.080	0.089	0.124	FALSE
	Meal regularity	0.396	0.000	0.000	TRUE

	Psychosocial resources	0.337	0.000	0.000	TRUE
	Disease count	-0.096	0.042	0.068	FALSE
	Stress eating	-0.189	0.000	0.000	TRUE
Screen time	Sleep duration	-0.108	0.022	0.040	TRUE
	Meals/day	0.112	0.017	0.033	TRUE
	Meal regularity	-0.145	0.002	0.005	TRUE
	Psychosocial resources	-0.105	0.026	0.044	TRUE
	Disease count	-0.041	0.385	0.461	FALSE
	Stress eating	0.160	0.001	0.002	TRUE
Sleep duration	Meals/day	0.062	0.188	0.246	FALSE
	Meal regularity	0.162	0.001	0.002	TRUE
	Psychosocial resources	0.167	0.000	0.001	TRUE
	Disease count	-0.121	0.010	0.021	TRUE
	Stress eating	-0.073	0.120	0.163	FALSE
Meals/day	Meal regularity	0.122	0.009	0.020	TRUE
	Psychosocial resources	-0.076	0.106	0.146	FALSE
	Disease count	0.086	0.067	0.100	FALSE
	Stress eating	0.082	0.084	0.119	FALSE
Meal regularity	Psychosocial resources	0.330	0.000	0.000	TRUE
	Disease count	-0.018	0.702	0.749	FALSE
	Stress eating	-0.120	0.011	0.023	TRUE
Psychosocial resources	Disease count	-0.127	0.007	0.015	TRUE
	Stress eating	-0.244	0.000	0.000	TRUE
Disease count	Stress eating	0.020	0.672	0.730	FALSE

Table S11. Group comparisons and multivariable associations for diet quality.

A. Group comparisons for DQI

**Comparison**	**Group 1 n**	**Group 1 DQI median [IQR]**	**Group 0 n**	**Group 0 DQI median [IQR]**	**Mann–Whitney U**	**Rank-biserial r**	**p**	**q-FDR**
Female vs male	373	16.17 [5.43–24.99]	77	10.97 [−2.99–21.19]	17108.5	0.191	0.008	0.014
Age ≥75 vs <75	174	12.84 [3.99–24.13]	276	15.97 [3.11–25.66]	22570.5	−0.060	0.283	0.283
Higher education vs other	291	16.86 [5.97–25.66]	159	11.43 [2.32–21.87]	26548.0	0.148	0.010	0.014
Multimorbidity vs none/one disease	62	9.89 [−1.30–16.03]	388	16.32 [5.42–25.84]	8217.0	−0.317	<0.001	<0.001
Supplement use vs none	261	16.17 [7.94–24.86]	189	13.94 [0.00–25.71]	27135.0	0.100	0.070	0.084
Regular meals vs irregular/sometimes	276	16.56 [8.20–26.84]	174	11.44 [0.04–22.52]	28084.0	0.170	0.002	0.007

B. Multi-group contrasts for DQI							
**Factor**	**No. of groups**	**Group summaries**	**Kruskal–Wallis H**	**df**	**Epsilon-squared**	**p**	**q-FDR**
Knowledge category	3	Good: 25.26 [15.83–30.76]; Insufficient: 5.00 [−6.49–17.14]; Sufficient: 16.17 [8.37–23.49]	40.50	2	0.086	<0.001	<0.001
Active ageing tertile	3	T1 low: 11.07 [−0.31–19.68]; T2 mid: 17.74 [11.43–24.99]; T3 high: 13.94 [2.81–26.88]	18.54	2	0.037	<0.001	<0.001
Meal regularity	3	No: 10.91 [−0.73–25.71]; Sometimes: 12.71 [3.99–20.54]; Yes: 16.56 [8.20–26.84]	9.23	2	0.016	0.010	0.013
Stress-related eating	3	No: 16.17 [8.37–27.46]; Sometimes: 13.94 [3.73–21.90]; Yes: 13.80 [0.00–23.49]	6.48	2	0.010	0.039	0.039

C. Adjusted multivariable associations with DQI				
**Predictor**	**OLS β (95% CI), HC3 robust SE**	**p**	**Logistic OR (95% CI)**	**p**
Female sex	5.24 (0.11 to 10.37)	0.046	2.62 (1.31 to 5.23)	0.006
Age (years)	0.14 (−0.12 to 0.40)	0.284	1.07 (1.02 to 1.11)	0.003
Higher education	−0.05 (−3.23 to 3.14)	0.976	1.31 (0.78 to 2.18)	0.305
Nutrition knowledge score	0.29 (−0.10 to 0.68)	0.144	1.10 (1.04 to 1.16)	<0.001
Physical activity score	1.55 (−0.40 to 3.49)	0.119	1.50 (1.14 to 1.98)	0.004
Cognitive activity score	0.00 (−1.72 to 1.73)	0.999	0.96 (0.75 to 1.23)	0.763
Screen time (hours/day)	−0.64 (−1.70 to 0.42)	0.238	1.00 (0.88 to 1.13)	0.973
Sleep score	3.66 (0.31 to 7.01)	0.033	1.84 (1.15 to 2.95)	0.012
Meal regularity	2.04 (−0.40 to 4.48)	0.102	0.97 (0.70 to 1.35)	0.870
Psychosocial resources	4.14 (−0.16 to 8.44)	0.059	1.61 (0.80 to 3.25)	0.180
Disease count	−2.84 (−5.37 to −0.32)	0.027	0.65 (0.44 to 0.96)	0.031

Notes: DQI values are presented as medians [IQR]. Binary comparisons were performed using the Mann–Whitney U test, with rank-biserial correlation reported as the non-parametric effect-size measure. Multi-group comparisons were performed using the Kruskal–Wallis test, with epsilon-squared reported as the effect-size measure. q-FDR values were calculated using the Benjamini–Hochberg procedure. Diagnostic assessment of the adjusted OLS model indicated non-normal residuals according to the Shapiro–Wilk test (W = 0.948, p < 0.001). The Breusch–Pagan test indicated heteroscedasticity (LM = 41.35, p < 0.001); therefore, HC3 heteroscedasticity-consistent robust standard errors were used for OLS inference. VIF values ranged from 1.09 to 1.41, indicating no relevant multicollinearity among predictors.

Table S12. Diagnostic assessment of the adjusted OLS model.

Diagnostic	Value	Interpretation
n	450	Complete cases used in OLS model
R^2	0.109	Model fit
Adjusted R^2	0.087	Model fit
Shapiro–Wilk W for residuals	0.948	Residual normality diagnostic
Shapiro–Wilk p for residuals	$p < 0.001$	$p < 0.05$ indicates non-normal residuals
Breusch–Pagan LM statistic	41.345	Heteroscedasticity diagnostic
Breusch–Pagan p value	0.000	$p < 0.05$ indicates heteroscedasticity
Breusch–Pagan F statistic	4.029	Heteroscedasticity diagnostic
Breusch–Pagan F p value	0.000	$p < 0.05$ indicates heteroscedasticity
VIF minimum (excluding intercept)	1.089	Multicollinearity diagnostic
VIF maximum (excluding intercept)	1.410	Values <5 indicate no relevant multicollinearity
Robust SE applied	Yes (HC3)	Applied because Breusch–Pagan $p < 0.05$

Table S13. Summary of identified senior profiles.

A. Sociodemographic, diet-quality, and knowledge profile

Profile	n	Age (years)	Female (%)	Higher education (%)	DQI (mean)	Healthy DQI (%)	Knowledge score	Active ageing score	Disease count
Health-engaged / higher-resource	335	72.81	84.8	76.7	16.69	26.3	12.83	0.269	0.964
Lower-resource / nutritionally vulnerable	115	76.02	77.4	29.6	10.85	17.4	7.34	-0.783	1.557

B. Health and lifestyle resource profile

Profile	Multimorbidity (%)	Abdominal obesity (%)	Supplement use (%)	Physical activity score	Cognitive activity score	Psychosocial resources (score)	Sleep score
Health-engaged / higher-resource	4.2	41.2	63.0	13.76	16.15	84.98	1.82
Lower-resource / nutritionally vulnerable	41.7	41.7	43.5	7.52	7.37	66.74	1.53

Notes: Values are presented as means or percentages for the two segmentation-derived profiles. Higher positive active-ageing values indicate a more favorable profile, whereas higher disease-count values indicate a greater burden of declared morbidity.

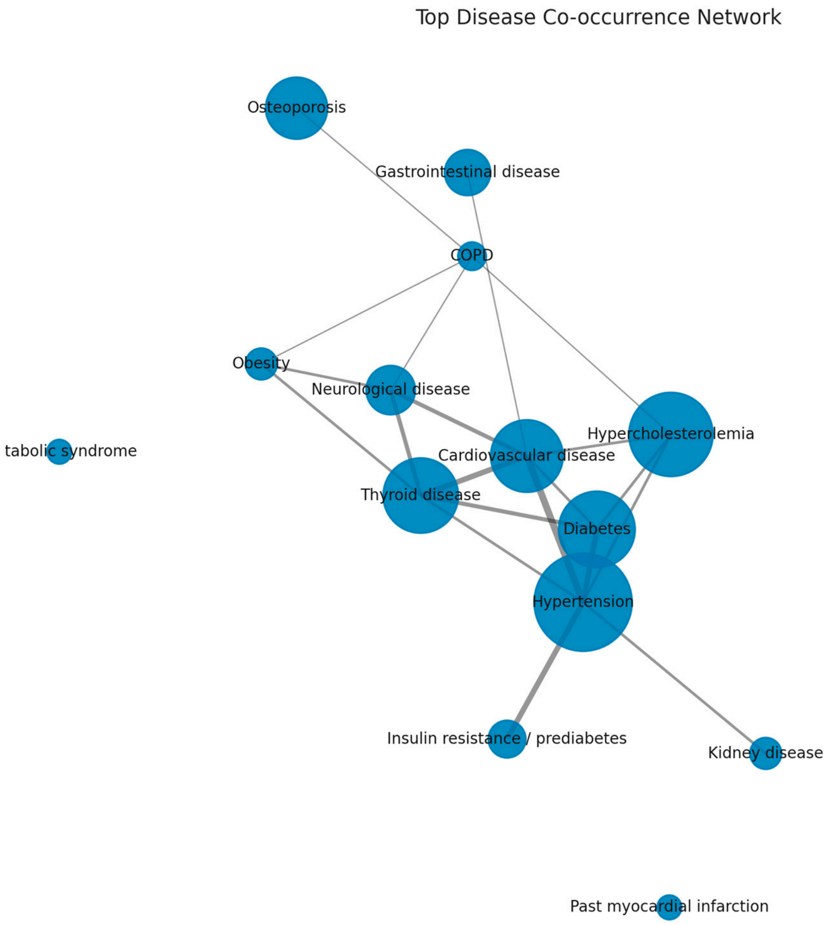

Figure S1. Disease co-occurrence network. The network visualizes co-occurring self-reported chronic conditions in the study population. Nodes represent disease categories, whereas connecting lines indicate their co-occurrence among respondents.

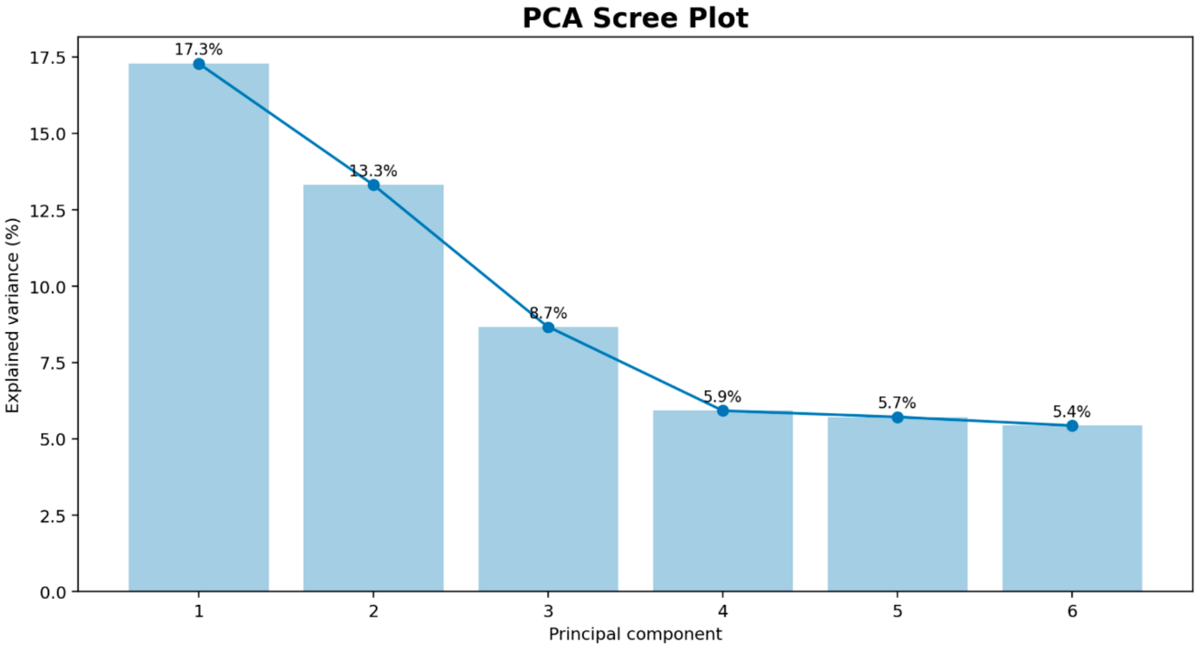

Figure S2. PCA scree plot. The scree plot presents eigenvalues of successive principal components and was used, together with explained variance and interpretability, to support component-retention decisions.

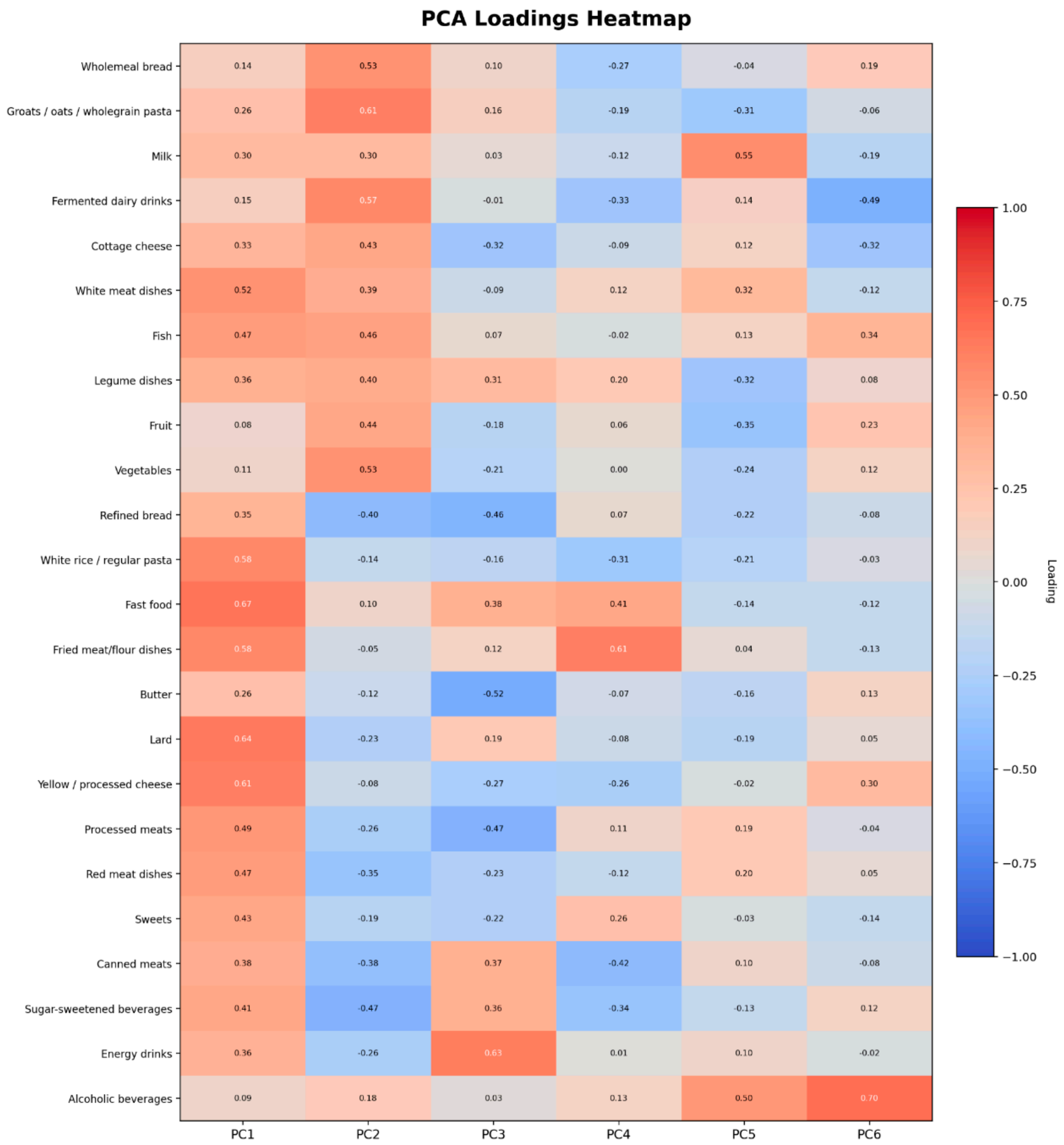

Figure S3. PCA loadings heatmap. The heatmap presents the rotated component loadings of food-frequency variables across the retained dietary patterns. Higher absolute values indicate stronger contributions of a given food group to a component.

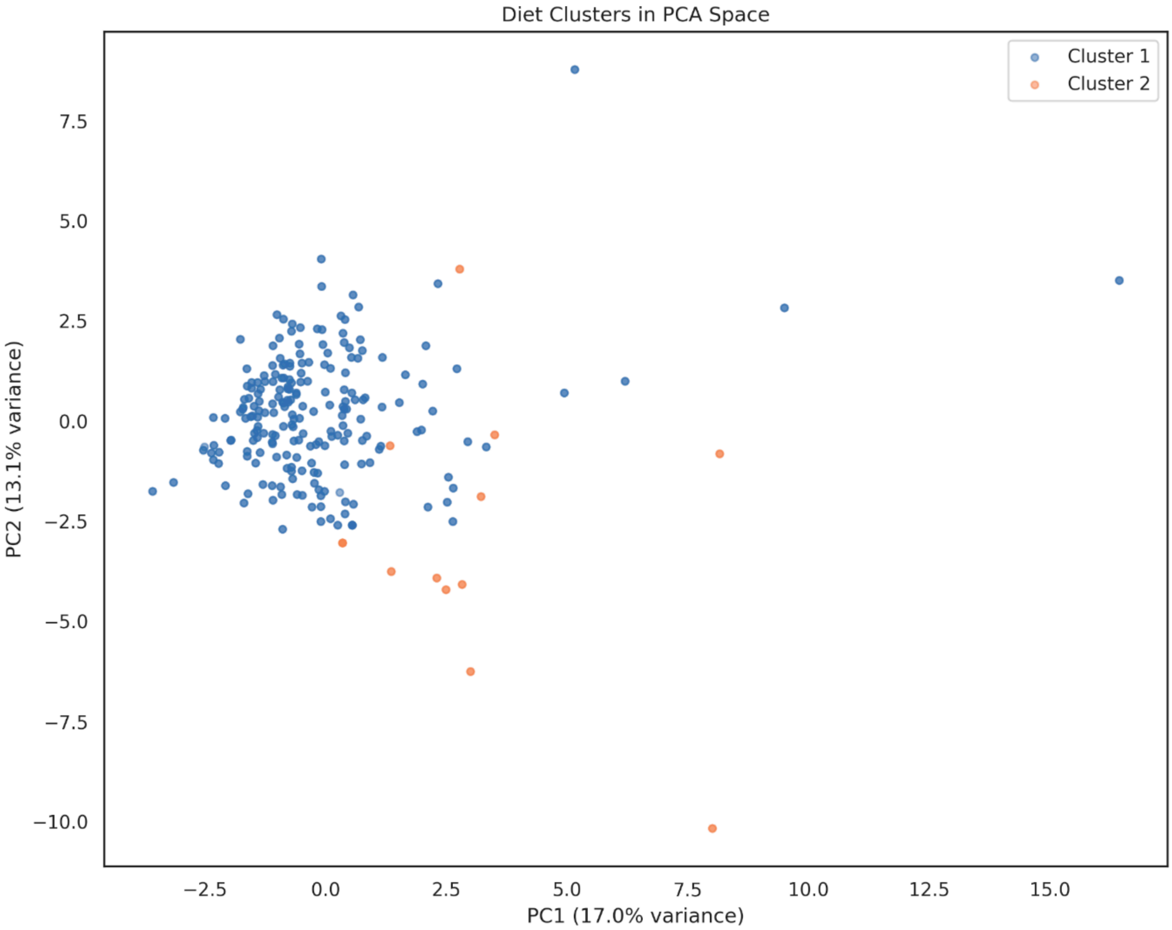

Figure S4. Diet clusters in PCA space. The plot shows the distribution of participants in the space defined by the first principal components and illustrates the exploratory clustering structure of dietary profiles.

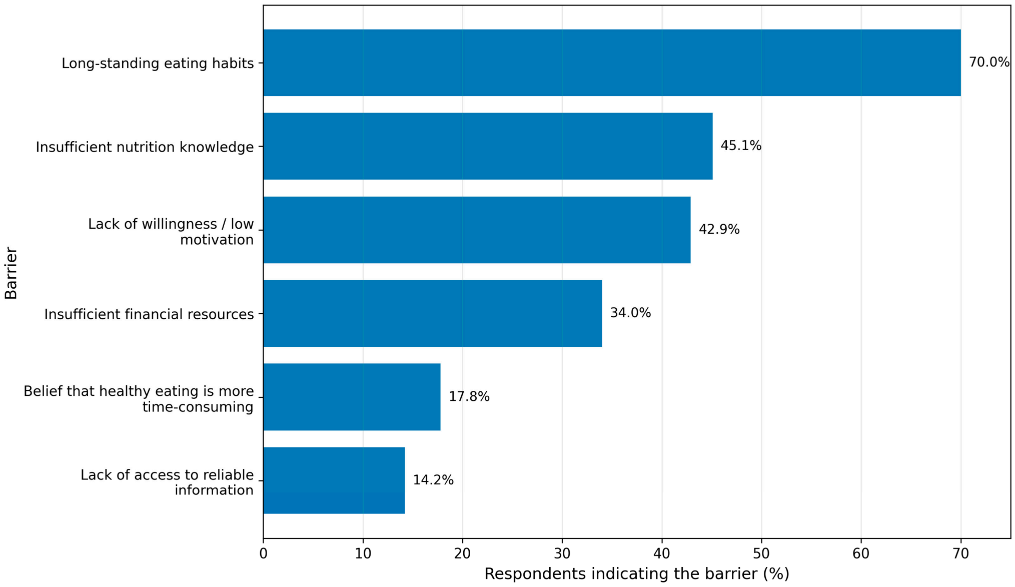

Figure S5. Barriers to healthy eating. The figure presents the frequency of self-reported barriers that may limit adherence to healthy eating principles among older adults attending U3A. Because respondents could select more than one answer, percentages do not sum to 100%.

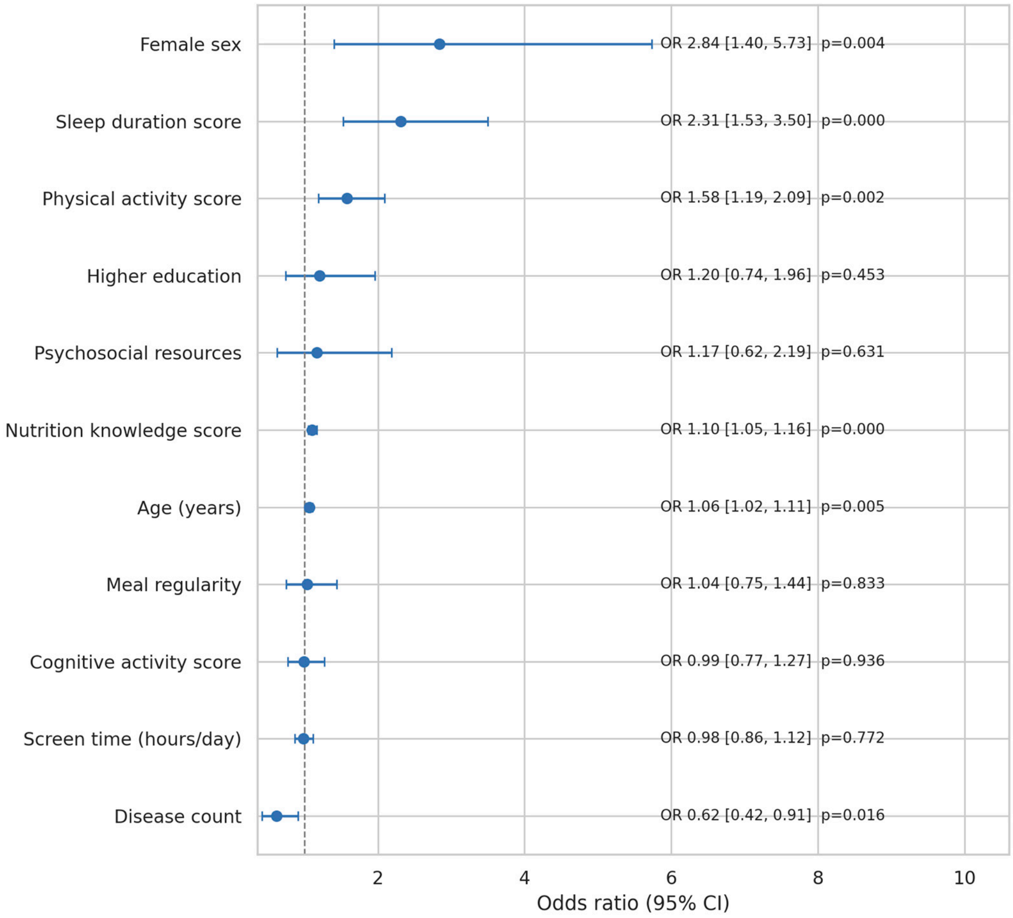

Figure S6. Predictors of higher diet quality (forest plot). The forest plot presents adjusted odds ratios and 95% confidence intervals from the logistic regression model for upper-tertile Diet Quality Index (DQI). Odds ratios above 1 indicate higher odds of better diet quality, whereas odds ratios below 1 indicate lower odds.

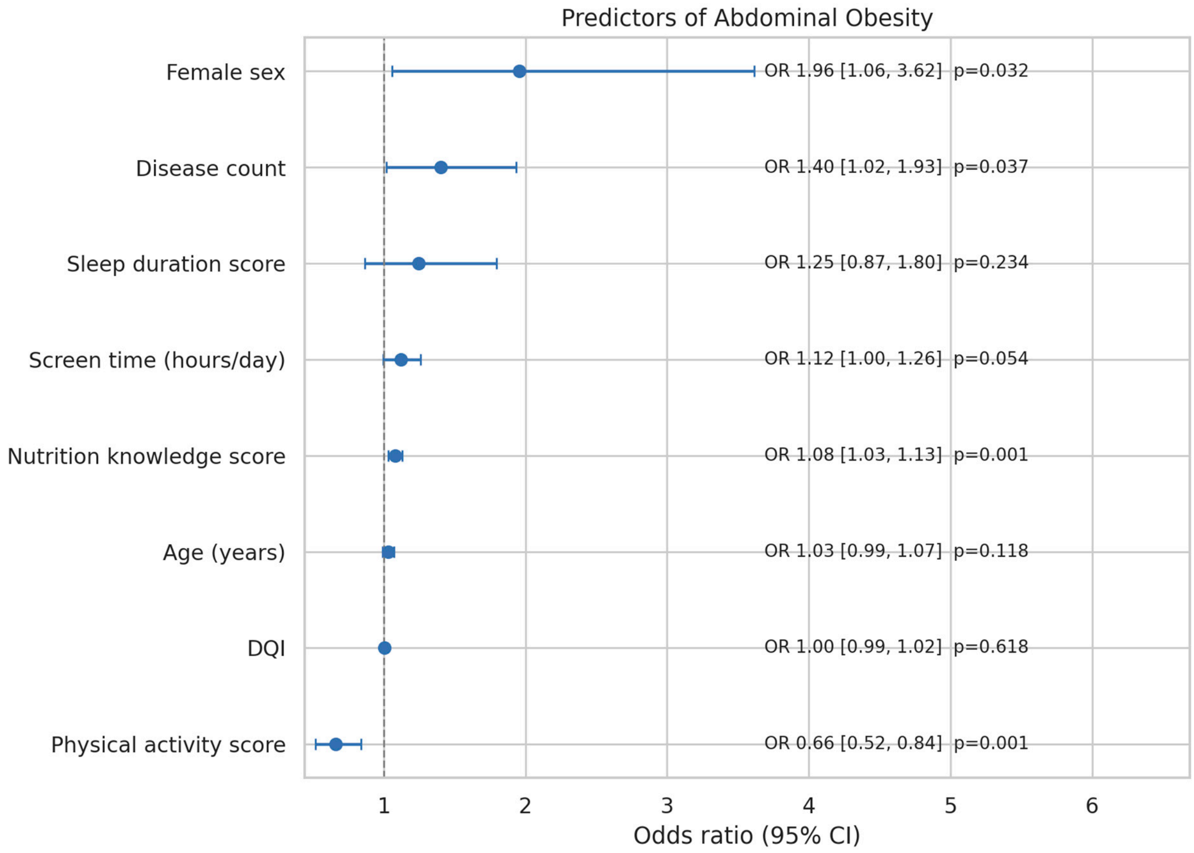

Figure S7. Predictors of abdominal obesity. The forest plot presents adjusted associations from the supplementary logistic regression model for abdominal obesity. Estimates above 1 indicate higher odds of abdominal obesity, whereas estimates below 1 indicate lower odds.

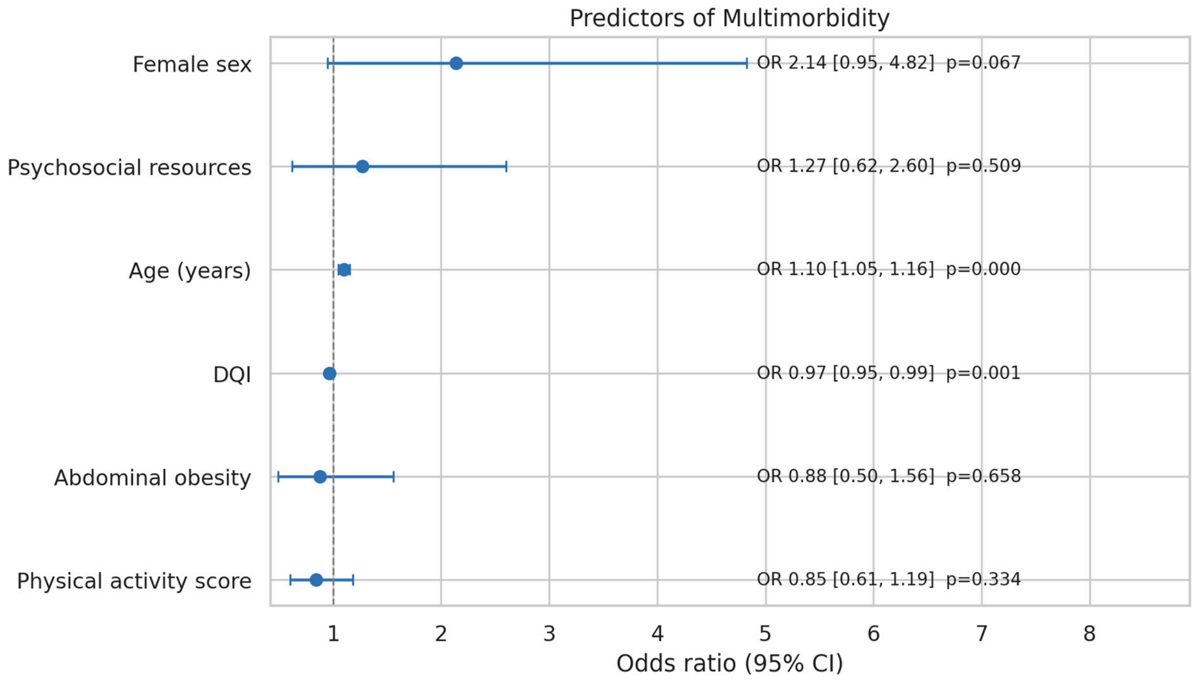

Figure S8. Predictors of multimorbidity. The forest plot presents adjusted associations from the supplementary logistic regression model for multimorbidity. Estimates above 1 indicate higher odds of multimorbidity, whereas estimates below 1 indicate lower odds.

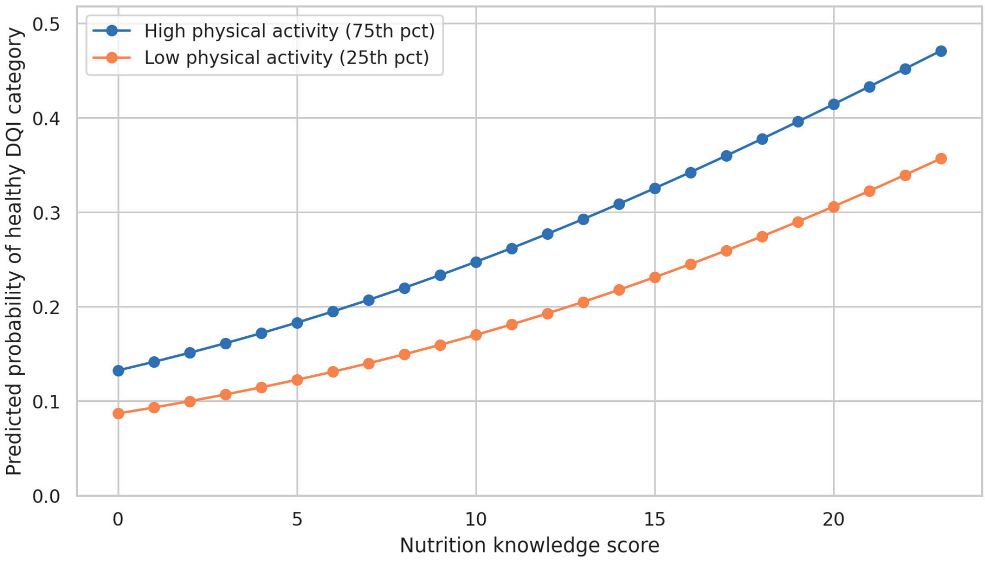

Figure S9. Predicted probability of healthy diet quality by nutrition knowledge and physical activity. The figure presents model-based predicted probabilities of achieving a healthy diet-quality profile across the range of nutrition knowledge scores, stratified by physical-activity level. The curves illustrate the combined association of nutrition knowledge and physical activity with the probability of healthier diet quality.

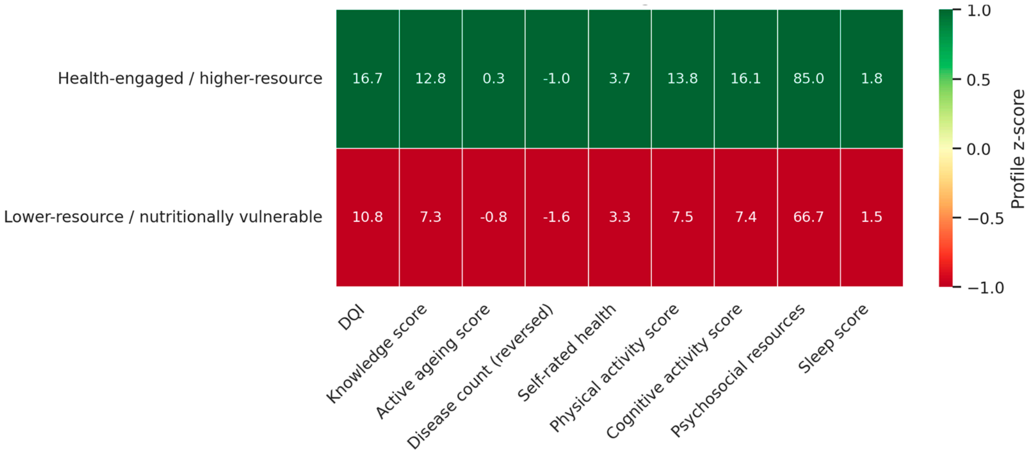

Figure S10. Senior profile segmentation heatmap. The heatmap presents standardized differences between the identified senior profiles across nutritional, lifestyle, psychosocial, and health-related variables. Higher values indicate a more favorable profile for positively oriented variables, whereas disease burden was interpreted in the opposite direction.

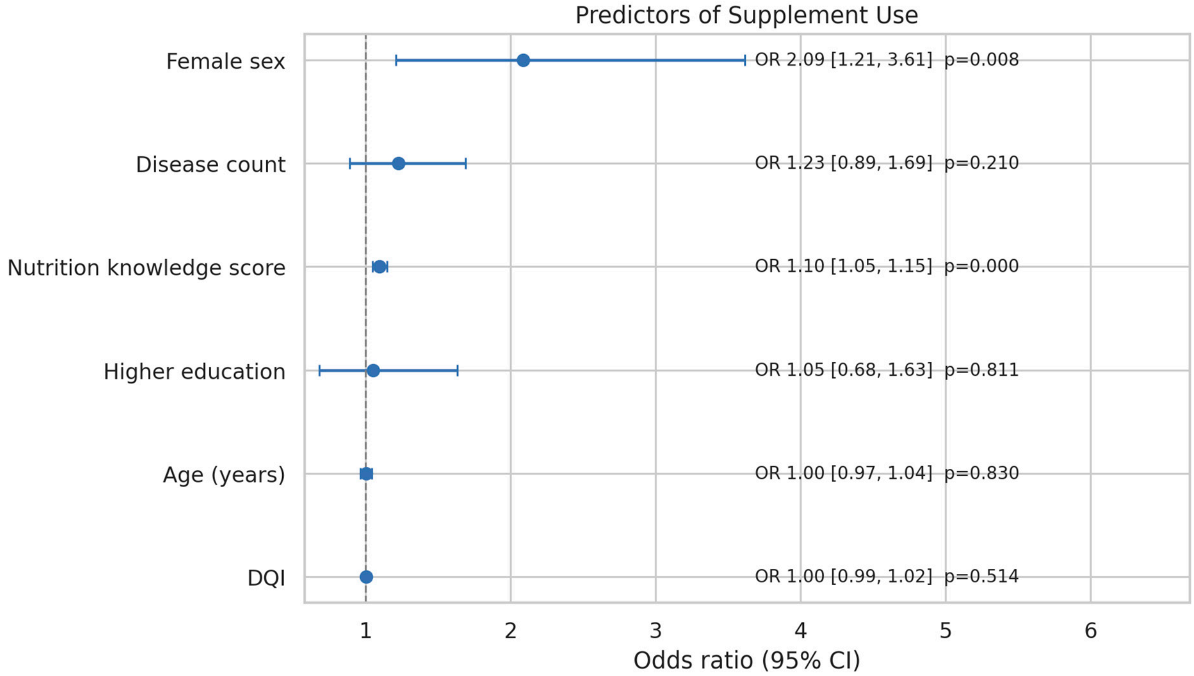

Figure S11. Predictors of supplement use. The forest plot presents adjusted associations from the supplementary logistic regression model for supplement use. Estimates above 1 indicate higher odds of supplement use, whereas estimates below 1 indicate lower odds.
